# Dissection of Allelic Variation Underlying Floral and Fruit Traits in Flare Tree Peony (*Paeonia rockii*) Using Association Mapping

**DOI:** 10.3389/fgene.2021.664814

**Published:** 2021-08-11

**Authors:** Xin Guo, Chunyan He, Fangyun Cheng, Yuan Zhong, Xinyun Cheng, Xiwen Tao

**Affiliations:** ^1^Beijing Advanced Innovation Center for Tree Breeding by Molecular Design, Beijing Forestry University, Beijing, China; ^2^Peony International Institute, School of Landscape Architecture, Beijing Forestry University, Beijing, China; ^3^Beijing Key Laboratory of Ornamental Plants Germplasm Innovation & Molecular Breeding, Beijing Forestry University, Beijing, China; ^4^Key Laboratory of Genetics and Breeding in Forest Trees and Ornamental Plants of Ministry of Education, Beijing Forestry University, Beijing, China; ^5^National Engineering Research Center for Floriculture, Beijing Forestry University, Beijing, China; ^6^Beijing Guose Peony Technology Co. Ltd., Beijing, China

**Keywords:** flare tree peony (*P. rockii*), SSR markers, floral and fruit traits, association mapping, linkage disequilibrium

## Abstract

Allelic variation in floral quantitative traits, including the elements of flowers and fruits, is caused by extremely complex regulatory processes. In the genetic improvement of flare tree peony (*Paeonia rockii*), a unique ornamental and edible oil woody species in the genus *Paeonia*, a better understanding of the genetic composition of these complex traits related to flowers and fruits is needed. Therefore, we investigated the genetic diversity and population structure of 160 *P. rockii* accessions and conducted single-marker association analysis for 19 quantitative flower and fruit traits using 81 EST-SSR markers. The results showed that the population had a high phenotypic diversity (coefficients of variation, 11.87–110.64%) and a high level of genetic diversity (mean number of alleles, *N*_A_ = 6.09). These accessions were divided into three subgroups by STRUCTURE analysis and a neighbor-joining tree. Furthermore, we also found a low level of linkage disequilibrium between these EST-SSRs and, by single-marker association analysis, identified 134 significant associations, including four flower traits with 11 EST-SSRs and 10 fruit traits with 32 EST-SSRs. Finally, based on the sequence alignment of the associated markers, P280, PS2, PS12, PS27, PS118, PS131, and PS145 may be considered potential loci to increase the yield of flare tree peony. These results laid the foundation for further analysis of the genetic structure of some key traits in *P. rockii* and had an obvious potential application value in marker-assisted selection breeding.

## Introduction

Flare tree peony (*Paeonia rockii*; FTP) is one of the most representative species in the *Paeonia* section *Moutan* that is native to China (Hong and Pan, 1999). Approximately 300 cultivars have been derived from this species, mainly distributed in northwest China, consisting of a large cultivar group that is distinct from the common tree peony (*Paeonia suffruticosa*) in China (Cheng et al., 2005; Cheng and Yu, 2008; Yuan et al., 2012). In addition to being cultivated as an ornamental species since the Tang Dynasty (618–906 AD), FTP has rapidly developed into a new woody edible oil plant in recent years as its seeds contain a high unsaturated fatty acid content (Li et al., 2015a,b; Zhang et al., 2017a,b; Wang et al., 2020). Therefore, improving ornamental quality and increasing the potential yield have become important objectives for FTP breeding. Among them, ornamental quality mainly includes floral traits, while yield is a complex character affected by multiple factors, in which the most important contributing factor is the fruit trait (Liu and Cheng, 2020b). Quantitative flower and fruit traits are controlled by multiple genes and have moderate to high heritability. As conventional breeding methods take more than 10 years to develop new FTP cultivars with stable comprehensive characteristics (Cheng, 2012), molecular breeding approaches have become inevitable selection methods to breed new tree peony cultivars (Gao et al., 2013; Zhou et al., 2015).

The development of DNA markers has provided broad prospects for accelerating the selection of intricate quantitative traits in trees, especially in tree peonies (Du et al., 2013; Yu et al., 2013; Wang et al., 2014; Resende et al., 2017). Quantitative trait locus (QTL) mapping studies have been carried out on parental populations and attempts have been made to use QTLs for breeding (Cai et al., 2015; Guo et al., 2017; Zhang et al., 2019). When used in selective breeding, substantial deficiencies in QTL mapping have been widely discussed (Bernardo, 2008; Grattapaglia and Resende, 2011). Due to the method’s low mapping ability, only a few QTLs hidden under target traits were found, and the variances explained by these QTLs were overestimated (Beavis, 1998). Association mapping based on linkage disequilibrium (LD), proposed as a way to deconvolute QTL mapping, can identify natural alleles for specific phenotypes, providing a valuable opportunity to shorten breeding years and improve breeding efficiency (Neale and Savolainen, 2004; Thumma et al., 2005). In molecular marker-assisted selection (MAS) breeding, expressed sequence tag (EST)-simple repeat sequence (SSR) markers are ideal choices because they are highly variable, codominant, and highly informative (Varshney et al., 2005). In FTP, a few associations between polymorphisms in EST-SSR and certain traits have been reported (Wu et al., 2017; Cui et al., 2018; Liu and Cheng, 2020a). Wu et al. (2017) employed single-marker association analysis on a sample of 462 individuals to identify 46 significant associations, including 37 EST-SSRs involving 11 flower traits, which explained 2.68–23.97% (mean 5.50%) of the phenotypic variation. Cui et al. (2018) found that 15 EST-SSRs were significantly associated with five oil-related traits based on association analysis of 205 individuals with phenotypic traits over three consecutive years. In an association analysis of 420 individuals, Liu et al. identified 141 significant associations involving 17 yield-related traits and 41 EST-SSRs, and the phenotypic variation was relatively small (mean 11.34%; Liu and Cheng, 2020a). To reduce environmental disturbances and measurement errors, asexual reproduction is often necessary to increase the accuracy of phenotypic measurements (Du et al., 2013). Although there have been previous studies on the association analysis of important traits in FTP, the samples used were all individuals without asexual reproduction; the core germplasm resources of FTP were not used.

In this study, 160 accessions (each containing three clones) from core germplasm resources of FTP and 81 EST-SSRs were used for single-marker association analysis to explore the allelic effects of floral and fruit trait variation. The results of this study laid a foundation for further identifying the key trait linkage loci of FTP, which is of great value to the genetic improvement of related traits.

## Materials and Methods

### Plant Materials

A total of 160 accessions (each containing three clones), representing the core germplasm resources of FTP that had been demonstrated and described by Guo et al. (2020), were used in this study. All the samples were originally introduced from Gansu Province in Northwest China, which was the main cultivation area, and had been randomly cultivated in the same nursery field of Beijing Guose Peony Garden in Beijing, China (40°45'N, 115°97'E) for more than 10 years. All the measured plants, approximately 15 years old, can produce stable flowers and fruits annually. The complete list of accessions used in this study is provided in Supplementary Table S1.

### Phenotyping

Seven flower quantitative traits, namely, flower diameter (FD), petal length (PL), petal width (PW), flare length (FL), flare width (FW), petal number (PN), and carpel number (CN), in the 160 accessions were measured at full bloom using digital calipers (YB5001B, Kraftwelle Industrial Co. Ltd., China) in May 2019. In addition, a total of 12 fruit quantitative traits, namely, the number of carpels with seeds (NCWS), multiple fruit fresh weight (MFFW), single fruit length (SFL), single fruit width (SFW), single fruit height (SFH), single fruit pericarp thickness (SFPT), multiple fruit seed number (MFSN), multiple fruit seed fresh weight (MFSFW), individual fruit number (IFN), individual seed number (ISN), individual fruit fresh weight (IFFW), and individual seed fresh weight (ISFW), were measured using digital calipers and electronic balances in September 2019. All measurements were carried out, as described in Supplementary Table S2. A total of nine flowers and nine fruits of each accession were collected, with three flowers and three fruits from one plant per replicate. The average value for each trait from three replicates for each accession was used for statistical and association analysis.

### DNA Extraction and SSR Marker Genotyping

The total genomic DNA of 160 accessions was extracted from fresh young leaves using a DNAsecure plant kit (Tiangen Biotech, Beijing, China), following the manufacturer’s instructions. The quality of the extracted DNA was determined by electrophoresis on 2% agarose gels and visualization using a UnicoUV-visible Spectrophotometer (Agilent, Palo Alto, CA, United States). Then, the DNA were diluted with deionized water to 20–30 ng/μl and stored in a freezer at –20°C.

The polymorphism of 140 previously developed SSR markers (Homolka et al., 2010; Hou et al., 2011; Gai et al., 2012; Zhang et al., 2012; Yu et al., 2013; Wu et al., 2014; Liu and Cheng, 2020a) was evaluated using a random sample of 12 accessions. After screening, a total of 81 EST-SSRs (Supplementary Table S3) were used to reveal the polymorphism of these 160 accessions. The SSR-PCR amplification reaction was conducted in a 10-μl solution, including 5 μl of 2×Power Taq PCR Master MIX (Aidlab Biotechnologies, Beijing, China), 3 μl of ddH_2_O, 1 μl of 20–25 ng/μl genomic DNA, and 0.5 μl of 10 μmol/L each of forward and reverse primers, and the procedure was performed as described by Wu et al. (2014). The PCR products were differentiated by capillary electrophoresis using an ABI3730xl DNA Analyzer (Applied Biosystems, Carlsbad, CA, United States). Micro-Checker 2.2.3 (Van Oosterhout et al., 2004) was applied to identify and correct genotyping errors.

### Data Analysis

The statistics software SPSS 18.0 (IBM Inc., Chicago, IL, United States; Davis, 2008) was used to analyze the average values of the investigated traits per accession. The coefficient of variation (CV) of each trait was calculated as follows: (standard deviation/mean) × 100%. The variation in 19 traits was estimated by one-way analysis of variance (ANOVA), and Pearson’s correlations between traits were calculated. Prior to ANOVA and Pearson’s correlation analysis, all SM data were tested for normality with the Shapiro-Wilk *W* test and for homogeneity of variance with Levene’s test, and the nonnormal data were logarithmically transformed. In addition, Benjamini-Hochberg (BH) FDR correction was used to correct the values of *p* for Pearson’s correlation analysis.

The capillary electrophoresis data were analyzed using GeneMarker 2.2.0. GenAIEx 6.5 (Peakall and Smouse, 2012) was used to calculate the following statistics: number of different alleles (*N*_A_), number of effective alleles (*N*_E_), Shannon’s information index (*I*), observed heterozygosity (*H*_o_), expected heterozygosity (*H*_e_), inbreeding coefficients (*F*_IS_), and Nei’s genetic diversity (GD). The polymorphism information content (PIC) of each locus was calculated using the Microsatellite Toolkit. GENEPOP 4.2 (Rousset, 2008) was used to detect microsatellite loci deviating from the Hardy-Weinberg equilibrium (HWE), and the results were applied to multiple tests with Bonferroni correction. Additionally, MEGA-X was used to construct a neighbor-joining (NJ) phylogenetic tree based on Nei’s unbiased genetic distance (Kumar et al., 2018).

The number of subpopulations (*K*) was detected by STRUCTURE 2.3.4 through an admixture model (Pritchard et al., 2000). First, the *K* value ranges from 1 to 10, and 10 independent operations were carried out for each *K* value, with a burn-in period of 100,000 times and 200,000 replications. Then, the results were uploaded to Structure Harvester (Earl and VonHoldt, 2012) to determine the final optimal *K* value. The optimum *K* value was inferred from Ln*P*(*D*) and Δ*K* (Evanno et al., 2005). For 10 repetitions of each *K* value, CLUMPP 1.1 was used to analyze the results from replicate analyses (Jakobsson and Rosenberg, 2007). Then, the outputs of each *K* value were visualized using CLUMPP and DISTRUCT (Rosenberg, 2004). The matrix corresponding to the *K* value of the optimal population structure was used for association analysis to correct false positives.

The degree of LD between loci was evaluated by the square of the allelic frequency (*r*^2^), which was calculated by using TASSEL 2.0.1. *r*^2^ = 0.1 was taken as the critical value to determine whether two loci had LD (*r*^2^ > 0.1: LD). Then, a mixed linear model (MLM) of TASSEL 2.0.1 was used to incorporate SSR data, phenotypic traits, *Q* matrix, and kinship matrix for association analysis. *Q* was the matrix of the optimal *K* value obtained through Structure Harvester. The kinship matrix was calculated by SPAGeDi 1.2 (Hardy and Vekemans, 2002). An adjusted value of *p* was employed for association analysis using false discovery rate (FDR) correction under QVALUE in *R* (Storey and Tibshirani, 2003). The ratio of dominance (*d*) to additive (*a*) effects was used to assess the gene effects of the significant loci obtained by association analysis. The boundaries of additive effect, partial to full dominance and overdominance, were |*d*/*a*| ≤ 0.50, 0.50 < |*d*/*a*| < 1.25, and |*d*/*a*| > 1.25, respectively. The calculation formulas of additive (*a*) effects and dominance (*d*) were as follows: 2*a* = |G_BB_ − G_bb_|; *d* = G_Bb_ − 0.5 (G_BB_ + G_bb_; G represents the average of phenotypes corresponding to genotype, BB and bb: homozygous genotypes, and Bb: heterozygous genotypes; Eckert et al., 2009).

Open Reading Frame Finder (ORF Finder) was used to find the complete ORFs of the associated marker sequences and translate the ORF sequences into protein sequences (Rombel et al., 2002). Then, the results were compared in the Arabidopsis Information Resource (TAIR) to find protein sequences with higher homology (Berardini et al., 2015). Finally, DNAMAN was used to construct a phylogenetic tree and protein sequence alignment map.

## Results

### Statistical and Correlation Analyses of Phenotypic Traits

As the tested samples can represent the current situation of FTP germplasm resources in China, ANOVA showed that the phenotypic variation range of all measured traits were wide (Table 1). The CVs ranged from 11.87% (FD) to 110.64% (ISFW), with an average of 48.01%. The CVs of fruit traits (mean 58.44%) were higher than those of flower traits (mean 30.13%), among them the traits ISFW (110.64%), PN (109.70%), and ISN (102.77%) were relatively higher. Correlation analysis of different traits showed 81 significant correlations (*p* < 0.05), of which 65 were very significant correlations (*p* < 0.01; Table 2). Considering the flower traits, excluding FL and PN, FW and CN, and PN and CN, all other traits were significantly correlated. Among the 66 correlation factors in fruit traits, 39 were significantly correlated. Especially when we considered ISFW as the yield index, MFFW, SFL, SFW, MFSN, MFSFW, IFN, ISN, and IFFW were very significantly positively correlated with ISFW, and the correlation coefficients were 0.726, 0.474, 0.599, 0.730, 0.820, 0.515, 0.960, and 0.814, respectively.

**Table 1:** Descriptive statistics of 19 quantitative traits in 160 flare tree peony (FTP) accessions.

Trait	Minimum	Maximum	Mean ± SD	Standard deviation	Coefficient of variation (%)	*F*
FD	9.67	19.67	15.40 ± 0.14	1.83	11.87	3.340^**^
PL	5.43	10.07	7.54 ± 0.07	0.90	11.93	0.810^**^
PW	4.68	9.93	7.42 ± 0.09	1.11	15.00	1.237^**^
FL	1.43	4.30	2.69 ± 0.05	0.57	21.26	0.327^**^
FW	0.67	3.72	1.98 ± 0.04	0.53	27.03	0.286^**^
PN	7.00	257.00	39.00 ± 3.38	42.78	109.70	1,829.992^**^
CN	4.00	10.33	5.33 ± 0.06	0.75	14.13	0.567^**^
NCWS	3.56	11.33	5.52 ± 0.08	1.05	19.02	1.100^**^
MFFW	5.93	83.83	21.48 ± 0.80	10.15	47.23	102.935^**^
SFL	14.93	67.32	40.49 ± 0.47	5.98	14.77	35.761^**^
SFW	9.89	22.26	15.33 ± 0.19	2.44	15.91	5.951^**^
SFH	3.34	98.85	15.15 ± 0.56	7.07	46.69	50.017^**^
SFPT	1.20	17.57	2.59 ± 0.10	1.29	49.78	1.667^**^
MFSN	0.11	52.00	12.28 ± 0.77	9.79	79.67	95.761^**^
MFSFW	0.05	28.26	5.56 ± 0.37	4.74	85.29	22.486^**^
IFN	2.50	74.00	29.12 ± 1.24	15.69	53.87	246.051^**^
ISN	0.93	2,119.79	368.61 ± 29.95	378.83	102.77	143,513.737^**^
IFFW	43.18	2,294.00	509.98 ± 30.50	385.85	75.66	148,883.795^**^
ISFW	0.43	960.84	170.24 ± 14.89	188.35	110.64	35,474.893^**^

** *p* < 0.01.

**Table 2:** The correlation analysis of 19 quantitative traits in 160 FTP accessions.

Trait	FD	PL	PW	FL	FW	PN	CN	NCWS	MFFW	SFL	SFW	SFH	SFPT	MFSN	MFSFW	IFN	ISN	IFFW	ISFW
FD	1.000																		
PL	0.680^**^	1.000																	
PW	0.634^**^	0.733^**^	1.000																
FL	0.339^**^	0.546^**^	0.405^**^	1.000															
FW	0.281^**^	0.342^**^	0.500^**^	0.659^**^	1.000														
PN	−0.158^*^	−0.233^**^	−0.370^**^	−0.103	−0.167^*^	1.000													
CN	0.172^*^	0.181^*^	0.230^**^	0.175^*^	0.154	0.103	1.000												
NCWS	0.096	0.064	0.085	0.097	0.053	0.234^**^	0.654^**^	1.000											
MFFW	0.235^**^	0.177^*^	0.217^**^	−0.073	0.021	−0.235^**^	−0.012	−0.035	1.000										
SFL	0.271^**^	0.220^**^	0.227^**^	0.044	0.114	−0.098	−0.168^*^	−0.182^*^	0.789^**^	1.000									
SFW	0.257^**^	0.185^*^	0.262^**^	−0.014	0.138	−0.322^**^	−0.067	−0.106	0.867^**^	0.703^**^	1.000								
SFH	0.077	0.010	0.068	−0.030	0.058	−0.119	0.061	−0.056	0.231^**^	0.263^**^	0.209^**^	1.000							
SFPT	−0.005	0.049	0.130	0.040	0.180^*^	−0.032	0.013	0.124	0.270^**^	0.216^**^	0.298^**^	0.044	1.000						
MFSN	0.137	0.030	0.076	−0.084	−0.067	−0.299^**^	−0.048	−0.097	0.677^**^	0.368^**^	0.588^**^	0.107	−0.007	1.000					
MFSFW	0.150	0.065	0.102	−0.127	−0.087	−0.273^**^	−0.051	−0.088	0.841^**^	0.533^**^	0.694^**^	0.160^*^	0.061	0.938^**^	1.000				
IFN	0.042	−0.078	−0.097	−0.175^*^	−0.119	0.034	−0.114	−0.002	0.140	0.117	0.119	−0.032	−0.056	0.072	0.113	1.000			
ISN	0.095	−0.014	0.021	−0.158^*^	−0.101	−0.233^**^	−0.066	−0.058	0.605^**^	0.363^**^	0.529^**^	0.093	−0.021	0.775^**^	0.772^**^	0.523^**^	1.000		
IFFW	0.176^*^	0.096	0.097	−0.144	−0.075	−0.192^*^	−0.097	−0.036	0.726^**^	0.581^**^	0.634^**^	0.140	0.148	0.460^**^	0.591^**^	0.681^**^	0.739^**^	1.000	
ISFW	0.100	0.011	0.035	−0.191^*^	−0.122	−0.220^**^	−0.070	−0.059	0.726^**^	0.474^**^	0.599^**^	0.131	0.029	0.730^**^	0.820^**^	0.515^**^	0.960^**^	0.814^**^	1.000

** *p* < 0.01;

* *p* < 0.05.

### Genetic Diversity

In total, 81 polymorphic EST-SSRs were used to evaluate the diversity of 160 accessions (Table 3). Then, 493 alleles were identified, ranging from 2 to 20 (*N*_A_). The *N*_E_ ranged from 1.006 to 8.516, with an average of 2.603. The *I* varied from 0.021 to 2.357 (mean 1.026). The mean values of *H*_O_ and *H*_E_ were 0.501 and 0.524, respectively. The mean *F*_IS_ of 81 SSRs was −0.439, among which 63 pairs were negative. In addition, the PIC values ranged from 0.006 to 0.871, with an average of 0.476. After Bonferroni multiple comparisons, 43 SSR sites deviated significantly from HWE. Therefore, these deviated sites were removed in the subsequent analysis and 38 SSR sites were ultimately used for subsequent population structure analysis and association mapping.

**Table 3:** Diversity information parameter at 81 SSRs in 160 FTP accessions.

Locus	*N*_A_	*N*_E_	*I*	*H*_o_	*H*_E_	*F*_IS_	PIC	*p*
PS2	4	1.268	0.403	0.225	0.211	−0.853	0.193	0.986^ns^
PS7	8	2.446	1.279	0.388	0.591	−0.064	0.558	0.000
PS8	6	2.884	1.172	0.738	0.653	−0.476	0.584	0.046^ns^
PS10	6	2.681	1.217	0.163	0.627	0.368	0.571	0.000
PS12	7	2.924	1.367	0.688	0.658	−0.387	0.624	0.303^ns^
PS17	4	2.118	0.829	0.513	0.528	−0.443	0.418	0.000
PS19	8	3.453	1.483	0.794	0.710	−0.407	0.673	0.090^ns^
PS21	20	8.516	2.357	0.925	0.883	−0.165	0.871	0.000
PS24	3	1.013	0.042	0.013	0.012	−0.992	0.012	1.000^ns^
PS25	10	3.234	1.480	0.406	0.691	0.103	0.648	0.000
PS27	6	1.422	0.589	0.275	0.297	−0.631	0.272	0.693^ns^
PS30	5	2.262	0.930	1.000	0.558	−1.235	0.460	0.000
PS31	7	2.390	1.053	0.519	0.582	−0.310	0.493	0.000
PS33	7	3.620	1.446	0.650	0.724	−0.174	0.678	0.000
PS36	3	1.730	0.628	0.513	0.422	−0.793	0.335	0.049^ns^
PS43	9	1.563	0.808	0.388	0.360	−0.716	0.342	1.000^ns^
PS46	4	1.284	0.416	0.200	0.221	−0.684	0.200	0.000
PS47	4	2.100	0.903	0.219	0.524	0.106	0.447	0.000
PS49	11	5.214	1.927	0.425	0.808	0.282	0.788	0.000
PS50	6	1.660	0.745	0.413	0.397	−0.641	0.352	0.326^ns^
PS53	4	2.661	1.050	1.000	0.624	−0.978	0.546	0.000
PS55	4	2.620	1.055	0.550	0.618	−0.271	0.550	0.000
PS56	6	3.402	1.321	1.000	0.706	−0.710	0.652	0.000
PS57	3	1.759	0.639	0.463	0.432	−0.640	0.341	0.741^ns^
PS59	4	1.322	0.436	0.281	0.243	−0.912	0.217	0.638^ns^
PS62	11	2.764	1.318	0.288	0.638	0.188	0.588	0.000
PS64	4	1.801	0.802	0.413	0.445	−0.483	0.392	0.226^ns^
PS66	6	2.132	1.045	0.113	0.531	0.319	0.485	0.000
PS73	12	7.077	2.101	0.819	0.859	−0.095	0.843	0.000
PS75	12	5.269	1.913	0.600	0.810	0.070	0.786	0.000
PS85	6	2.438	1.107	0.650	0.590	−0.512	0.538	0.643^ns^
PS90	9	4.010	1.729	0.563	0.751	0.001	0.726	0.000
PS91	4	2.247	1.043	0.544	0.555	−0.425	0.511	0.764^ns^
PS93	4	2.606	1.040	1.000	0.616	−1.007	0.540	0.000
PS94	6	2.762	1.162	0.463	0.638	−0.087	0.568	0.235^ns^
PS96	13	3.993	1.714	0.800	0.750	−0.318	0.723	0.000
PS97	5	1.181	0.371	0.163	0.153	−0.906	0.149	1.000^ns^
PS98	4	2.076	0.782	0.494	0.518	−0.434	0.402	0.948^ns^
PS102	5	3.381	1.306	1.000	0.704	−0.716	0.651	0.000
PS103	8	4.111	1.612	0.700	0.757	−0.168	0.721	0.000
PS105	2	1.098	0.189	0.094	0.089	−0.960	0.085	0.534^ns^
PS113	4	2.050	0.760	1.000	0.512	−1.440	0.393	0.000
PS114	10	4.047	1.557	0.494	0.753	0.097	0.711	0.000
PS116	5	2.376	1.108	0.263	0.579	0.126	0.538	0.000
PS117	5	2.791	1.213	0.288	0.642	0.194	0.597	0.000
PS118	3	2.061	0.860	0.563	0.515	−0.578	0.444	0.327^ns^
PS122	4	1.494	0.634	0.138	0.331	−0.085	0.305	0.000
PS123	3	1.918	0.687	0.781	0.478	−1.154	0.367	0.000
PS129	5	2.223	0.953	0.113	0.550	0.346	0.473	0.000
PS131	4	2.517	1.020	0.588	0.603	−0.372	0.527	0.077^ns^
PS142	6	1.515	0.720	0.056	0.340	0.174	0.320	0.000
PS145	6	2.535	1.127	0.663	0.606	−0.488	0.547	0.892^ns^
PS147	11	4.610	1.787	0.838	0.783	−0.286	0.755	0.000
PS151	12	6.593	2.047	0.456	0.848	0.310	0.831	0.000
PS159	12	2.639	1.527	0.350	0.621	0.058	0.602	0.000
PS160	9	3.615	1.607	0.656	0.723	−0.184	0.691	0.000
PS163	11	4.922	1.753	0.838	0.797	−0.254	0.767	0.000
P002	7	1.536	0.794	0.131	0.349	−0.027	0.335	0.000
P026	4	1.712	0.637	0.431	0.416	−0.621	0.334	0.986^ns^
P061	4	2.024	0.731	0.525	0.506	−0.532	0.384	0.895^ns^
P067	6	2.570	1.224	0.494	0.611	−0.197	0.573	0.000
P068	5	3.046	1.281	0.750	0.672	−0.445	0.620	0.056^ns^
P138	3	2.023	0.726	0.650	0.506	−0.780	0.384	0.152^ns^
P150	3	2.540	0.999	0.594	0.606	−0.373	0.527	0.785^ns^
P162	3	1.253	0.384	0.188	0.202	−0.728	0.185	0.672^ns^
P180	3	1.246	0.384	0.206	0.197	−0.848	0.182	0.871^ns^
P221	5	2.538	1.035	0.656	0.606	−0.477	0.524	0.000
P235	3	1.589	0.631	0.375	0.370	−0.642	0.320	0.080^ns^
P242	2	1.013	0.038	0.013	0.012	−0.994	0.012	0.937^ns^
P260	2	1.479	0.505	0.356	0.324	−0.777	0.271	0.204^ns^
P265	3	1.733	0.650	0.481	0.423	−0.715	0.341	0.234^ns^
P280	7	2.844	1.368	0.669	0.648	−0.383	0.614	0.422^ns^
P281	2	1.006	0.021	0.006	0.006	−0.997	0.006	0.968^ns^
P290	4	1.703	0.689	0.513	0.413	−0.829	0.348	0.000
P296	5	1.963	0.891	0.481	0.491	−0.491	0.431	0.964^ns^
P318	8	3.820	1.448	0.844	0.738	−0.405	0.691	0.430^ns^
P333	5	1.429	0.580	0.281	0.300	−0.637	0.274	0.400^ns^
Seq6	7	1.683	0.777	0.450	0.406	−0.703	0.360	0.000
50F, R	12	3.960	1.713	0.775	0.748	−0.289	0.721	0.000
5F, R	5	1.187	0.365	0.169	0.158	−0.911	0.152	0.999^ns^
PCA1	5	2.509	1.069	1.000	0.601	−1.061	0.524	0.000

Significant level for deviations from Hardy-Weinberg equilibrium (significance is *p* < 0.01). ns, not significant.

### Population Structure

**Figure 1:**
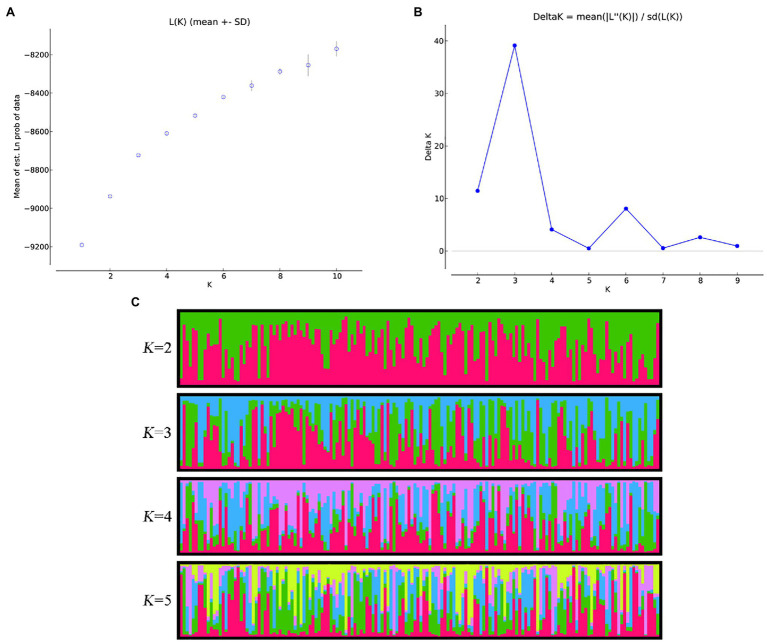
Estimation of genetic structure of 160 accessions using 38 simple repeat sequences (SSRs) based on the STRUCTURE. **(A)** Log probability data [Ln*P*(*D*)] for each *K* value (10 replicates). **(B)** Δ*K* estimates of the posterior probability distribution of the data for a given *K*. **(C)** Estimated population structure and clustering of 160 accessions with *K* = 2–5. Accessions are shown by thin vertical lines.

**Figure 2:**
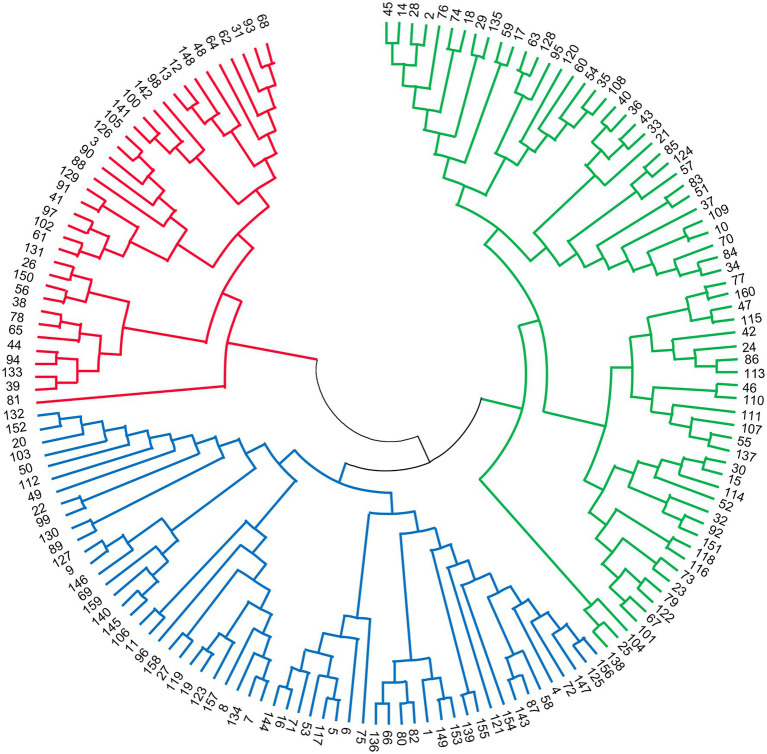
The Neighbor-Joining (NJ) tree of 160 accessions based on the data of 38 SSRs.

Through STRUCTURE analysis of 160 accessions with 38 EST-SSRs, as the *K* value increased, Ln*P*(*D*) progressively increased overall (Figure 1). When *K* reached 3, the rising trend of Ln*P*(*D*) slowed down. Moreover, the Δ*K* value corresponding to the maximum *K* value was the population structure; therefore, the 160 accessions could be divided into three subgroups. The outputs of *K* = 3 were visualized through CLUMPP and DISTRUCT, and all samples showed a wide range of mixed lineages. Then, we visualized the outputs from *K* = 2–5 and found that each sample was highly heterozygous. The *Q* matrix output of the three subgroups can be used for structure-based association analysis. The phylogenetic tree divided these samples into three major clades (Figure 2), revealing similar clustering results to those obtained using STRUCTURE. In summary, the two methods classified these samples into three subgroups.

### LD Level

**Figure 3:**
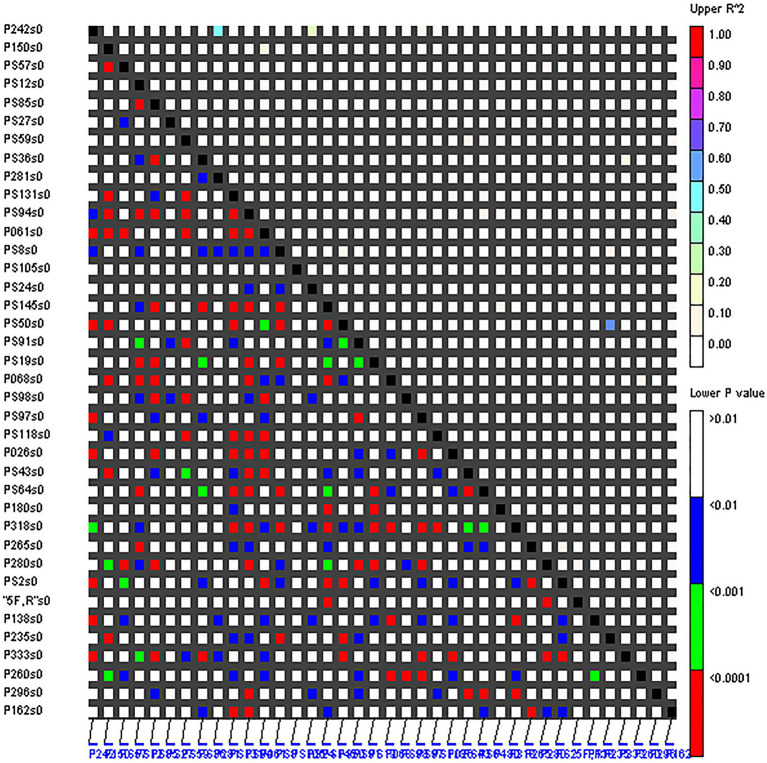
Pairwise linkage equilibrium (LD; *r*^2^) between 38 SSRs. The *x*- and *y*-axes represent the 38 SSRs, *r*^2^ < 0.1 represents linkage equilibrium, and the different colors correspond to the thresholds of *r*^2^ and *p*.

The LD levels of 38 EST-SSRs in the 160 accessions were evaluated (Figure 3). Based on the *r*^2^ estimates, only 0.71% (*r*^2^ ≥ 0.1) of the loci sites had significant LD. In all 698 locus pairs (*r*^2^ < 0.1), 17.62, 30.23, and 50.14% of the locus pairs displayed linkage equilibrium at *p* < 0.05, *p* < 0.01, and *p* < 0.001, respectively. Therefore, the overall LD level of 38 EST-SSRs in the 160 accessions was low, and most loci were in linkage equilibrium with each other. However, there were also several loci with significant LD levels between them, such as marker P235-PS50 (*r*^2^ > 0.3; *p* < 0.001).

### Single-Marker Associations of Floral Traits

For flower traits, 266 (38 EST-SSRs × 7 traits) single-marker associations were detected by the MLM model, and a total of 21 (7.89%) significant associations between four floral traits and 14 EST-SSRs were detected under the condition of *p* < 0.01. Then, FDR test was carried out on these 21 significantly associated combinations (*Q* < 0.05), and 17 significantly associated combinations of four flower traits and 11 EST-SSRs were ultimately identified, with an explainable rate of 2.23–26.34% (mean 11.80%; Table 4). The statistical results of the gene effects indicated that 2 (11.76%) associated combinations presented additive effects, 2 (11.76%) presented partial to full dominance, and 13 (76.47%) presented overdominance.

**Table 4:** Significant SSR marker-trait pairs from the association test results of flower traits.

Trait	Locus	*p*	*Q*-value	*R*^2^ (%)	2*a*	*d*	*d*/*a*	2*a*/*s*_p_
FD	P26	2.00E−06	7.60E−05	2.23	0.0405	0.2332	11.5136	0.0221
	PS19	1.15E−03	2.19E−02	26.34	1.2277	0.5450	0.8878	0.6714
PL	P26	1.13E−03	4.28E−02	11.02	0.0096	0.1294	26.9583	0.0168
	PS24	2.93E−03	3.71E−02	7.16	1.0545	−2.1573	−4.0915	1.8448
	P235	3.53E−03	3.35E−02	10.69	0.3987	0.3841	1.9265	0.6975
	PS8	3.96E−03	3.01E−02	14.6	0.3015	−0.0402	−0.2663	0.5275
	P281	4.34E−03	2.75E−02	5.03	1.6221	−3.4890	−4.3018	2.8378
	PS97	5.05E−03	2.74E−02	9.08	0.1067	−2.7200	−50.9831	0.1867
	P333	5.59E−03	2.65E−02	11.12	0.1372	−0.1193	−1.7391	0.2400
	PS91	8.27E−03	3.49E−02	12.53	0.0055	0.0513	18.6364	0.0096
PW	P281	9.29E−04	3.53E−02	6.72	1.7530	−2.8435	−3.2442	3.2801
	PS24	1.70E−03	3.24E−02	7.8	0.7014	−1.6207	−4.6213	1.3124
	P235	5.26E−03	5.00E−02	10.14	0.4548	0.2835	1.2467	0.8510
	PS57	5.34E−03	4.06E−02	7.79	0.1996	0.0357	0.3577	0.3735
	PS43	6.90E−03	4.37E−02	14.68	0.0872	0.2411	5.5287	0.1632
CN	P26	2.00E−06	7.60E−05	18.26	0.0615	0.1042	3.3870	0.0817
	PS19	1.15E−03	2.19E−02	25.38	0.0320	0.4916	30.7250	0.0425

*p*, significance level for association (significance is *p* < 0.01); *R*^2^, percentage of the phenotypic variance explained; *Q*-value, a correction for multiple testing [false discovery rate FDR (*Q*) < 0.05]; *s*_p_, standard deviation for the phenotypic trait under consideration.

The number of significantly associated combinations for each trait ranged from two (FD) to eight (FL). There were two markers that were significantly related to FD and CN, and the highest interpretation rates were found for locus PS19, which were 26.34 and 25.38%, respectively. There were eight EST-SSRs that were significantly related to FL, with interpretation rates of 5.03–12.53% (mean 10.15%), and the highest explanatory rate was locus PS91. Among them, there were one and seven combinations with additive effects and overdominance, respectively. There were five EST-SSRs that were significantly related to FW, with explanatory rates ranging from 6.72 to 14.68% (mean 9.43%), and the highest interpretation rate was locus PS43. No significant associations with PL, PW, and PN were detected in this study. Additionally, five EST-SSRs were markedly associated with more than one trait. For instance, P26 was significantly related to FD, FL, and CN, while P235, P281, and PS24 were significantly associated with FL and FW.

### Single-Marker Associations of Fruit Traits

For fruit traits, 456 (38 EST-SSRs × 12 traits) single-marker associations were detected by the MLM model, and a total of 118 (25.88%) significant associations between 10 fruit traits and 32 EST-SSRs were detected under the condition of *p* < 0.01. Then, an FDR test was carried out on these 118 significantly associated combinations (*Q* < 0.05), and 117 significantly associated combinations of 10 fruit traits and 32 EST-SSRs were ultimately identified, with explanatory rates of 0.18–43.40% (average 17.68%; Table 5). The statistical results of gene effects showed that 13 (11.11%) associated combinations appeared to have additive effects, 28 (23.93%) appeared to have partial to full dominance, and 76 (64.96%) appeared to have overdominance.

**Table 5:** Significant SSR marker-trait pairs from the association test results of fruit traits.

Trait	Locus	*p*	*Q*-value	*R*^2^ (%)	2*a*	*d*	*d*/*a*	2*a*/*s*_p_
NCWS	P26	7.17E−07	2.73E−05	19.63	0.0240	−0.0310	−2.5833	0.0229
	PS19	1.12E−04	2.13E−03	28.87	0.3633	0.6151	3.3859	0.3465
MFFW	P242	3.89E−09	1.48E−07	19.76	40.4683	−41.2109	−2.0367	3.9887
	PS24	3.99E−10	7.59E−09	24.09	62.7752	−52.4424	−1.6708	6.1873
	PS59	4.07E−10	5.16E−09	25.79	2.9592	−21.8023	−14.7353	0.2917
	PS2	6.90E−10	6.55E−09	26.8	62.3351	−33.4870	−1.0744	6.1439
	PS145	1.61E−08	1.23E−07	31.98	4.5925	−0.2254	−0.0982	0.4527
	PS85	1.70E−08	1.08E−07	31.93	0.7140	1.8037	5.0524	0.0704
	P318	3.68E−08	2.00E−07	34.1	2.5125	−1.7301	−1.3772	0.2476
	PS131	4.25E−08	2.02E−07	26.53	1.2683	−0.9481	−1.4950	0.1250
	PS27	4.74E−08	2.00E−07	25.19	3.2202	−0.2004	−0.1245	0.3174
	PS91	8.90E−08	3.38E−07	26.91	6.1555	−1.2124	−0.3939	0.6067
	PS12	2.80E−05	9.67E−05	30.77	2.2168	−1.4778	−1.3333	0.2185
	P280	6.70E−05	2.12E−04	28.73	2.9589	1.5406	1.0413	0.2916
	P162	2.70E−04	7.89E−04	11.45	23.4680	−13.0793	−1.1146	2.3131
	PS50	6.85E−04	1.86E−03	16.13	23.1626	−14.0836	−1.2161	2.2830
	P260	2.00E−03	5.06E−03	7.61	15.0406	−10.1388	−1.3482	1.4824
SFL	P318	5.54E−08	2.11E−06	33.66	0.4855	−0.2852	−1.1747	0.0812
	PS2	1.10E−05	2.09E−04	16.53	26.6660	−14.2622	−1.0697	4.4588
	PS24	1.20E−05	1.52E−04	13.44	27.0421	−53.7990	−3.9789	4.5217
	PS131	3.50E−05	3.33E−04	18.88	1.8602	−0.3113	−0.3347	0.3110
	PS85	6.90E−05	5.24E−04	22.24	5.8173	2.4138	0.8299	−0.9727
	PS59	7.70E−05	4.88E−04	12.92	0.4488	−40.4321	−180.1787	0.0750
	PS145	8.70E−05	4.72E−04	21.93	1.8258	−0.1060	−0.1161	0.3053
	PS27	1.20E−04	5.70E−04	16.23	4.9738	2.0351	0.8183	0.8317
	PS43	1.21E−04	5.11E−04	20.5	25.3766	12.7560	1.0053	4.2432
	P61	3.36E−04	1.28E−03	12.52	0.6538	−0.5210	−1.5938	0.1093
	PS12	1.17E−03	4.05E−03	25.35	3.6484	−2.0494	−1.1235	0.6100
	P242	4.03E−03	1.28E−02	5.11	12.1354	−46.4023	−7.6474	2.0291
SFW	P242	2.69E−04	1.02E−02	8.08	6.2226	−18.3637	−5.9023	2.5511
	PS91	1.47E−03	2.79E−02	15.07	1.8603	0.1002	0.1078	0.7627
	PS2	2.95E−03	3.73E−02	9.78	6.8639	−4.1569	−1.2112	2.8140
MFSN	P242	1.26E−11	4.78E−10	5.53	20.6461	−22.3470	−2.1648	2.1098
	P150	1.45E−08	2.75E−07	17.77	2.1017	−6.8179	−6.4879	0.2148
	PS57	2.00E−06	2.53E−05	9.43	0.7912	−6.2323	−15.7540	0.0809
	PS12	3.00E−06	2.85E−05	29.76	4.4831	−2.3336	−1.0410	0.4581
	PS85	3.00E−06	2.28E−05	17.62	9.3424	2.4571	0.5260	0.9547
	PS27	3.00E−06	1.90E−05	4.67	2.5904	−2.9028	−2.2412	0.2647
	PS59	9.00E−06	4.89E−05	12.82	6.5719	−13.6332	−4.1489	0.6716
	PS36	1.10E−05	5.23E−05	11.36	2.6301	−7.5893	−5.7711	0.2688
	P281	1.20E−05	5.07E−05	0.18	5.2508	−14.8746	−5.6657	0.5366
	PS131	2.80E−05	1.06E−04	18.9	2.6919	−1.9727	−1.4656	0.2751
	PS94	3.50E−05	1.21E−04	15.52	5.5730	−3.9375	−1.4131	0.5695
	P61	5.90E−05	1.87E−04	11.22	3.5347	−5.9028	−3.3399	0.3612
	PS8	8.20E−05	2.40E−04	19.99	6.2574	−8.0737	−2.5805	0.6394
	PS105	8.40E−05	2.28E−04	11.81	11.4994	−16.9536	−2.9486	1.1751
	PS24	2.91E−04	7.37E−04	4.12	24.5754	−24.3823	−1.9843	2.5113
	PS145	5.12E−04	1.22E−03	32.1	0.2785	−5.1720	−37.1415	0.0285
	PS50	8.13E−04	1.82E−03	23.24	15.8575	−8.3573	−1.0540	1.6204
	PS91	9.54E−04	2.01E−03	22.89	3.7400	−6.8927	−3.6859	0.3822
	PS19	1.44E−03	2.88E−03	21.49	6.8567	7.4117	2.1619	0.7007
	P68	1.88E−03	3.56E−03	19.51	18.8487	−8.2082	−0.8710	1.9261
	PS98	2.49E−03	4.51E−03	12.63	3.6896	−5.9812	−3.2422	0.3770
	PS97	2.76E−03	4.77E−03	16.59	13.7810	−17.7528	−2.5764	1.4083
	PS118	4.61E−03	7.61E−03	17.99	1.2030	−8.3587	−13.8964	0.1229
	P26	7.19E−03	1.14E−02	9.2	2.6599	−6.4400	−4.8422	0.2718
	PS43	8.84E−03	1.34E−02	14.04	7.9873	3.5050	0.8776	0.8162
MFSFW	P242	4.01E−07	1.52E−05	15.05	16.5058	−13.6071	−1.6488	3.4808
	P280	1.36E−10	2.59E−09	43.4	3.6135	0.0060	0.0033	0.7620
	PS145	3.68E−09	4.66E−08	33.51	0.5759	−1.6869	−5.8581	0.1214
	PS91	2.59E−08	2.46E−07	28.22	1.8375	−2.6293	−2.8618	0.3875
	PS59	8.72E−08	6.62E−07	20.39	2.5827	−6.0147	−4.6576	0.5447
	PS131	8.96E−07	5.67E−06	23.2	0.4428	−0.7498	−3.3866	0.0934
	PS24	4.00E−06	2.17E−05	14.67	22.8577	−16.8312	−1.4727	4.8204
	P318	4.00E−06	1.90E−05	28.81	1.8230	−0.8516	−0.9343	0.3844
	PS2	5.00E−06	2.11E−05	17.55	22.8615	−11.7372	−1.0268	4.8212
	PS50	7.00E−06	2.66E−05	21.96	10.9331	−6.1514	−1.1253	2.3056
	PS85	1.00E−05	3.45E−05	24.67	3.0100	1.1058	0.7348	0.6348
	PS12	1.40E−05	4.43E−05	31.7	0.9607	−1.0020	−2.0859	0.2026
	PS118	4.10E−05	1.20E−04	15.06	1.3036	−3.9239	−6.0201	0.2749
	PS64	4.30E−05	1.17E−04	16.27	4.2022	0.7440	0.3541	0.8862
	PS27	2.69E−04	6.81E−04	15.21	0.4414	−1.1026	−4.9959	0.0931
	PS8	8.01E−04	1.90E−03	16.93	2.8796	−2.7622	−1.9185	0.6073
	PS36	8.43E−04	1.88E−03	10.08	2.3534	−3.8258	−3.2513	0.4963
	P162	9.78E−04	2.06E−03	9.9	10.7435	−5.3716	−1.0000	2.2657
	P150	1.22E−03	2.45E−03	12.1	1.1925	−3.0146	−5.0558	0.2515
	PS105	1.53E−03	2.91E−03	6.17	4.0299	−7.1978	−3.5722	0.8499
	PS97	1.67E−03	3.02E−03	10.51	4.9218	−7.4541	−3.0290	1.0379
	P260	1.75E−03	3.02E−03	7.77	6.3885	−4.7610	−1.4905	1.3472
	PS57	6.33E−03	1.05E−02	7.58	0.8793	−2.8582	−6.5010	0.1854
IFN	P150	2.56E−04	9.73E−03	14.1	6.9075	−3.8298	−1.1089	0.4404
ISN	P280	3.67E−04	1.39E−02	26.29	195.6839	96.1550	0.9828	0.5165
	P150	7.50E−04	1.43E−02	12.73	130.2499	−238.2099	−3.6577	0.3438
	PS118	9.46E−04	1.20E−02	11.23	20.7663	−235.6122	−22.6918	0.0548
	PS91	1.09E−03	1.04E−02	15.48	80.4876	−227.2271	−5.6463	0.2125
	PS64	2.65E−03	2.02E−02	11.07	175.7995	155.3601	1.7675	0.4641
	PS59	5.70E−03	3.61E−02	7.71	173.9827	−403.9357	−4.6434	0.4593
	PS12	6.83E−03	3.71E−02	22.36	208.7671	−32.2896	−0.3093	0.5511
	P242	9.67E−03	4.59E−02	4.16	693.3875	−706.6413	−2.0382	1.8303
IFFW	PS85	1.77E−09	6.71E−08	34.25	102.9559	−122.7455	−2.3844	0.2668
	PS24	3.40E−05	6.46E−04	12.28	1,672.3147	−1,333.3727	−1.5946	4.3341
	PS59	6.30E−05	7.98E−04	13.16	85.4445	−521.1641	−12.1989	0.2214
	PS27	2.73E−04	2.59E−03	15.19	210.2070	10.6415	0.1012	0.5448
	P242	3.11E−04	2.36E−03	7.93	974.6330	−985.1185	−2.0215	2.5259
	PS2	4.02E−04	2.55E−03	12.3	1,666.0573	−867.6269	−1.0415	4.3178
	P318	4.72E−04	2.56E−03	22.41	252.5740	−40.4510	−0.3203	0.6546
	PS131	4.79E−04	2.28E−03	15.56	12.9643	−78.5108	−12.1119	0.0336
	PS91	7.68E−04	3.24E−03	15.97	132.2274	−110.9912	−1.6788	0.3427
	PS145	1.58E−03	6.02E−03	17.87	98.3069	−44.8050	−0.9115	0.2548
	PS12	2.69E−03	9.30E−03	23.98	21.5857	−14.9735	−1.3873	0.0559
	P150	4.62E−03	1.46E−02	10.32	177.2492	−174.8099	−1.9725	0.4594
	P280	9.06E−03	2.65E−02	21.04	318.1074	2.3626	0.0149	0.8244
ISFW	P242	2.80E−05	1.06E−03	10.55	548.9454	−437.8473	−1.5952	2.9145
	PS59	3.40E−05	6.46E−04	13.87	67.6135	−181.3082	−5.3631	0.3590
	PS91	4.60E−05	5.83E−04	19.67	43.3480	−93.1077	−4.2958	0.2301
	P280	8.60E−05	8.17E−04	28.37	69.8530	51.2509	1.4674	0.3709
	PS24	9.30E−05	7.07E−04	11.16	795.7008	−562.9896	−1.4151	4.2246
	PS85	1.56E−04	9.88E−04	21.15	22.8575	10.7868	0.9438	0.1214
	PS2	2.76E−04	1.50E−03	12.77	796.2987	−404.4477	−1.0158	4.2278
	PS12	3.13E−04	1.49E−03	27.38	73.4092	−6.9552	−0.1895	0.3898
	PS131	6.79E−04	2.87E−03	15.09	7.6998	−29.8505	−7.7536	0.0409
	PS64	2.16E−03	8.22E−03	11.35	42.4772	85.9071	4.0449	0.2255
	PS145	2.26E−03	7.80E−03	17.34	20.6608	−64.2305	−6.2176	0.1097
	P318	2.39E−03	7.57E−03	19.95	120.9243	−30.5276	−0.5049	0.6420
	P150	2.55E−03	7.45E−03	11.13	68.8411	−111.4819	−3.2388	0.3655
	PS118	3.01E−03	8.16E−03	9.75	23.1819	−120.6926	−10.4127	0.1231
	PS27	3.04E−03	7.71E−03	11.98	25.3010	−24.1708	−1.9107	0.1343

*p*, significance level for association (significance is *p* < 0.01); *R*^2^, percentage of the phenotypic variance explained; *Q*-value, a correction for multiple testing [false discovery rate FDR(*Q*) < 0.05]; *s*_p_, standard deviation for the phenotypic trait under consideration.

The number of significantly associated combinations for each trait ranged from 1 (IFN) to 25 (MFSN). Of these, IFN had one significant association; NCWS had two significant associations; SFW had three significant associations; ISN had eight significant associations; SFL had 12 significant associations; IFFW had 13 significant associations; MFFW and IFSW had 15 significant associations each; MFSFW had 23 significant associations; and MFSN had 25 significant associations. We also found that in ISN, ISFW, and MFSFW, the interpretation rates of P280 were the highest, while in MFFW and SFL, the interpretation rates of P318 were the highest. No locus significantly associated with SFPT and SFH was detected in this study. The phenomenon in which one marker was significantly associated with multiple traits was more common in this part of the study. In the correlation analysis of fruit traits, ISN, IFFW, and ISFW were all significantly correlated with each other. In the association analysis results, six markers were significantly associated with these three traits at the same time, namely, loci P150, P242, P280, PS12, PS59, and PS91. Similarly, loci P242, PS12, PS131, PS145, PS24, PS27, PS50, PS59, PS85, and PS91 were significantly associated with MFSN, MFFW, and MFSFW.

There were 10 marker-trait combinations with explanatory rates exceeding 30%, including the following: MFFW associated with PS145, PS85, P318, and PS12; SFL associated with P318; MFSN associated with PS145; MFSFW associated with P280, PS145, and PS12; and IFFW associated with PS85. Through sequence alignment *via* the National Center for Biotechnology Information (NCBI), the gene sequences of P280, PS12, and PS145 were related to fruit and seed development in other species. The explanatory rate of P280 with MFSFW was the highest, at 43.4%, which was predicted to be *WRINKLED Like 1* (*WRIL1*) belonging to the basal *ANT* subgroup of the *APETALA2/ETHYLENE-RESPONSIVE ELEMENT-BINDING FACTOR* (*AP2/ERF*) family *AINTEGUMENTA* (*ANT*) group. This gene, which has a typical AP2 domain, has a similarity of 44.79% with AtWRIL1 in *Arabidopsis*, and its main function is now known to regulate lipid biosynthesis (Supplementary Figure S1; Kong et al., 2020; Zhai et al., 2021). After comparison, PS145 had no similarity with the genes studied in the database, but the protein sequences that were translated had WD40 domains, which can control seed weight and volume in *Arabidopsis* (You et al., 2011). The predicted results of PS12 showed that it has the highest similarity with *MYB5* of the MYB gene family in *Arabidopsis* and is mainly involved in seed coat development and oil biosynthesis (Supplementary Figure S2; Li et al., 2009, 2020; Cheng et al., 2021). In addition, PS2 and PS131 were also significantly associated with fruit traits in Liu’s research (Liu and Cheng, 2020a). PS2 was associated with MFFW, SFL, SFW, and MFSFW, and the explanation rate of MFFW was the highest, at 26.8%. This site has a MADS-box functional domain, and the study of the similar *AtSEP3* sequence in *Arabidopsis* showed that it controlled carpel and ovule development (Supplementary Figure S3; Liljegren et al., 2000; Favaro et al., 2003; Renard et al., 2020). The predicted result of PS131 was *WRKY*22 of the WRKY gene family, which can mediate dark-induced leaf senescence in *Arabidopsis* (Supplementary Figure S4; Zhou et al., 2011; Hsu et al., 2013; Gaudinier et al., 2018). Furthermore, *WRKY*22 (PS131), *GATA*8 (PS118; Supplementary Figure S5), and *ERF*3 (PS27; Supplementary Figure S6) jointly participate in nitrogen metabolism to regulate growth and development in *Arabidopsis* (Dubois et al., 2015; Taylor-Teeples et al., 2015; Gaudinier et al., 2018); whether these genes affect fruit and seed development in FTP through nitrogen metabolism needs further research. Therefore, the abovementioned markers provide important references for the molecular breeding of FTP.

## Discussion

### Genetic Diversity of the Associated Population

The selection of populations with high genetic diversity and molecular markers with high polymorphism is crucial for association analysis (Ingvarsson, 2008). Here, 19 quantitative traits and 81 SSRs were utilized to evaluate the diversity of 160 accessions. In previous studies of phenotypic variation of FTP, Pang et al. (2012) reported that the CVs of 32 traits in 150 individuals ranged from 10 to 30%, while Wu et al. (2017) found that the CVs of 29 quantitative traits in 462 individuals ranged from 9.52 to 112.1%. In addition, Liu et al. studied 24 quantitative traits of 420 individuals and found that the CVs ranged from 12.03 to 106.63% (Liu and Cheng, 2020a). This study revealed CVs ranging from 11.87 to 110.64% for 19 quantitative traits in 160 accessions, which was consistent with the results of Wu et al. and Liu et al. Because the sampling strategies of these three studies were essentially the same, all the associated populations were constructed through the screening of a large number of FTP genotypes, reflecting the genetic background and structure of cultivated FTP to a certain extent. Additionally, the phenotypic variation of the 160 accessions in this study was similar to the CVs of other studies, indicating that the associated population composed of 160 accessions has high phenotypic diversity.

Phenotypic variation analysis allowed us to obtain a basic understanding of the associated population, but phenotypic variation was easily affected by the environment, and some individuals exhibited small differences in phenotypic variation, which was difficult to distinguish only by phenotypic traits. Therefore, it was very important to use molecular markers to analyze genetic diversity and population structures. In this study, 81 SSRs detected a total of 493 alleles in 160 accessions, and the *N*_A_ was 6.09, which was larger than 40 SSRs in 462 individuals (*N*_A_ = 4.5; Wu et al., 2017) and 34 SSRs in 282 cultivars (*N*_A_ = 5.441; Guo et al., 2020), but smaller than 12 SSRs in 335 individuals of wild *P. rockii* (*N*_A_ = 9.15; Yuan et al., 2012). This suggested that, on the one hand, the SSR primers and plant materials used in this study had comparatively higher allele variation, and on the other hand, the diverse primers and materials used in the studies gave rise to discrepancies. Additionally, the mean *F*_IS_ of 81 SSRs was −0.439, among which 63 pairs were negative, demonstrating the existence of a heterozygote surplus in the 160 accessions. We speculated that this was correlated with the hybridization origin and self-incompatibility of tree peony, which was in accordance with the reported results (Yuan et al., 2014; Zhou et al., 2014). Simultaneously, compared with other outcrossing woody plants, the genetic diversity of this species was at a moderate level (PIC = 0.476; Botstein et al., 1980), which was higher than that of *Populus tomentosa* (Du et al., 2012), but inferior to that of *Prunus avium* (Ganopoulos et al., 2011).

### LD and Population Structure

The level of LD of the association population was a prerequisite for association analysis. There was a low level of LD between different SSR markers in this study. In general, the LD levels of outcrossing woody plants were low (Kulheim et al., 2009; Chhetri et al., 2019), and tree peony was also an outcrossing species so the LD levels of FTP in other studies were also low (Wu et al., 2017; Cui et al., 2018; Liu and Cheng, 2020a). Moreover, we speculated that many human interventions, such as selective breeding, were also important contributors to the low LD levels. However, the mechanism of the LD level was still not clear for this associated population because the distances of these loci on chromosomes were unclear. Additionally, low LD levels at a small number of SSR sites did not represent the level of the entire genome or the intergenomic region. Therefore, it is necessary to use multiple markers distributed throughout the whole genome to determine the LD level in one population, because different types of markers may provide different types of insight depending on their characteristics (Neale and Savolainen, 2004).

In addition to considering the LD level of the associated population, we should also pay attention to the genetic structure of the population, because in practical research, for various reasons, it is impossible to have a population without population structure. The effect of sample structure in populations used for genetic association studies has been well documented and identified as the cause of some false associations (Newman et al., 2001; Kang et al., 2010; Sul et al., 2018). Predicting the genetic structure of the population was the premise of association analysis, which can improve the accuracy and avoid the appearance of false positives as much as possible (King et al., 2010). SSR loci that deviate from HWE may indicate genotyping error, inbreeding, population subdivision, or selection (Balding, 2006). In this study, after the Bonferroni correction for multiple testing, 43 loci were found to deviate significantly from HWE, and they were excluded. Then, the two methods were utilized to evaluate population structure and produced coincident consequences. Through STRUCTURE analysis, the 160 accessions were divided into three subgroups, and to a large extent, this was supported by the NJ tree analysis, which was similar to previous reports of three subgroups in FTP (Wu et al., 2017; Guo et al., 2020). Furthermore, Yuan et al. (2012) revealed that 335 wild *P. rockii* individuals were mainly divided into three subpopulations, which were strongly linked to the geographical distribution pattern of wild *P. rockii*. We hypothesized that the three subgroups prescribed in this research correspond to the same three gene banks that were previously reported and also reflect their geographic origins.

Even if the subgroup structure of the associated population was considered, false positives could not be completely controlled by the general linear model (GLM; Sun et al., 2015). MLM has been demonstrated to be effective in controlling false positives and can effectively control the error rate due to its consideration of the population structure matrix (*Q*) and the kinship matrix (*K*; Yu et al., 2006). To further improve the accuracy of the association analysis results, FDR correction was carried out for all values of *p* of associations, greatly reducing the expansion of values of *p*. In this study, we found 139 significant associations, but this number dropped to 134 after FDR correction, akin to previous studies (Wu et al., 2017; Liu and Cheng, 2020a).

### Associations With Floral and Fruit Traits in Flare Tree Peony

In this study, 11 SSRs were demonstrated to be significantly associated with four floral traits, four of which were derived from the transcriptome sequences of tree peony flower buds (Wu et al., 2014), and a total of 117 significant associations of 32 SSR markers related to 10 fruit traits were identified. More than two loci were significantly associated with each trait, indicating that quantitative traits were controlled by microeffect polygenes. Complex quantitative traits of plants for association analysis can be significantly associated with many sites (Sun et al., 2015); similar conclusions have been reported in other studies of trees (Dillon et al., 2012; Porth et al., 2013). Simultaneously, we also found that a marker was significantly associated with multiple traits, such as P26, which was associated with FD, FL, and CN, which may be due to the significant correlation between these phenotypic traits, and may reflect the characteristics of pleiotropism (Thudi et al., 2014).

Phenotyping is an important part of tree association analysis. Typical associated populations are usually composed of different unrelated individuals grown under the same ambient conditions and augment what is known about the measurement of phenotypes, which must usually be asexually reproduced to reduce environmental interference and measurement errors (Du et al., 2013). Although there have been previous studies on the association analysis of important traits in FTP, the samples used were all individuals and had no asexual reproduction (Wu et al., 2017; Cui et al., 2018; Liu and Cheng, 2020a). Hence, in this study, we used 480 phenotypes (160 genotypes × 3 clones) to compensate for the limited number of EST-SSR markers, and repeated data from each accession could be integrated to generate a phenotypic mean for analysis, which reduced the impact of measurement errors. In this study, the average explanatory rates of floral and fruit traits were 11.80 and 17.68%, respectively, higher than those of Wu et al. (2017; flower traits: 5.50%) and Liu and Cheng (2020a; fruit traits: 6.53%), which also reflected the improvement of the effectiveness of association mapping.

Additionally, replication of genotype-phenotype associations is crucial in association mapping to distinguish false-positive associations. Therefore, of the 38 SSRs used in this study, 17 markers were in conformity with Wu et al. (2017), but the association analysis results of flower traits in the two studies were inconsistent. This may be due to differences in sample size, gene-environment interactions, genetic background, gene-gene interactions, or other factors; therefore, some real associations may not be repeated in unrelated datasets (Greene et al., 2009; Beaulieu et al., 2011; Du et al., 2013). In addition, there were 21 markers identical to those described in Liu et al. Most of the associations were different, but associations between the same markers and traits were still found in the two studies. In both studies, PS2 was significantly associated with MFFW, PS12 was significantly associated with MFFW and MFSFW, and PS131 was significantly associated with SFL (Liu and Cheng, 2020a). We hypothesized that these repeated associations might help identify important genomic regions. These findings were also of great value in the use of marker-assisted selective breeding for trait improvement. Future studies will require multiple germplasm populations to combine multiyear and multiplot phenotypic data to validate the developed markers.

Flare tree peony has developed into an emerging woody oil crop in China, and its output mainly refers to ISFW. PS12, PS27, PS131, PS118, and ps280 were significantly associated with ISFW, and may be the key sites affecting the yield. Studies have shown that the *MYB5* gene of PS12 is involved in seed coat development to control seed size and affect yield (Li et al., 2008; Su et al., 2011; Dong et al., 2017). The oil content in tree peony seeds can reach 27–33%, which can directly affect seed weight (Cui et al., 2016). The *WRIL1* gene corresponding to P280 may be an important gene regulating oil synthesis in tree peony. Nitrogen metabolism is closely related to plant growth and development, thereby affecting yield (Tilman et al., 2002). In this study, *GATA8* (PS118), *ERF3* (PS27), and *WRKY22* (PS131) may form a network to participate in nitrogen metabolism. In addition, *WRKY22* was also involved in leaf senescence. Relevant studies have shown that the combination of related genes can delay the senescence process of plants and significantly increase yield (Distelfeld et al., 2014; Lira et al., 2017). This showed that these loci were potential genes to increase the yield of FTP, which is worthy of further study.

The analysis of the genetic regulatory relationships between different significant association sites of the same quantitative trait was helpful for breeding with significant association combinations. For example, in floral traits, FD was an important ornamental character that determined its ornamental value to a large extent. P26 was significantly associated with FD and showed an overdominant effect (*d*/*a* = 11.5136), indicating that individuals with P26 heterozygosity sites may produce flowers of larger diameter. For fruit traits, ISFW was considered a yield indicator, with 15 markers significantly associated with it. Among them, 11 markers, namely, P242, PS59, PS91, P280, PS24, PS131, PS64, PS145, P150, PS118, and PS27, showed the same pattern of gene action (overdominance), while the effects of P280 (*d*/*a* = 1.4674) and PS64 (*d*/*a* = 4.0449) were positive, showing that individuals with P280 and PS64 heterozygous loci might generate heavier seeds. In summary, marker combinations based on the gene effect value of associated loci can be used for the early selection of target traits.

## Data Availability Statement

The datasets presented in this study can be found in online repositories. The names of the repository/repositories and accession number(s) can be found in the article/Supplementary Material.

## Author Contributions

XG and FC: conceptualization and validation. XG: formal analysis, data curation, writing – original draft preparation, and visualization. XG, XC, and XT: investigation. XC and XT: resources. XG, CH, FC, YZ, XC, and XT: writing – review and editing. FC: supervision and project administration. All authors contributed to the article and approved the submitted version.

## Conflict of Interest

XC and XT are employees of Beijing Guose Peony Technology Co. Ltd., Beijing, China.

The remaining authors declare that the research was conducted in the absence of any commercial or financial relationships that could be construed as a potential conflict of interest.

## Publisher’s Note

All claims expressed in this article are solely those of the authors and do not necessarily represent those of their affiliated organizations, or those of the publisher, the editors and the reviewers. Any product that may be evaluated in this article, or claim that may be made by its manufacturer, is not guaranteed or endorsed by the publisher.
