# Supplementary material for: Dissection of Allelic Variation Underlying Floral and Fruit Traits in Flare Tree Peony (Paeonia rockii) Using Association Mapping
Source: Front Genet. 2021 Aug 11;12:664814. doi: 10.3389/fgene.2021.664814 (PMC8385368; doi:10.3389/fgene.2021.664814)
Supplement: Supplementary file 1 [file Table_1.DOCX]

Supplementary Material

# Supplementary Tables

**Supplementary Table 1.** The names of 160 accessions used in this study.

| **Number** | **Name** |
| --- | --- |
| 1 | ‘Ao Yun Sheng Huo’ |
| 2 | ‘Bai Gui Fei’ |
| 3 | ‘Bai Zhang Bing’ |
| 4 | ‘C2’ |
| 5 | ‘C3’ |
| 6 | ‘C3-2’ |
| 7 | ‘C3-4’ |
| 8 | ‘C6’ |
| 9 | ‘C7’ |
| 10 | ‘Da Xiong Mao’ |
| 11 | ‘Fei Fen He’ |
| 12 | ‘Fen He’ |
| 13 | ‘Fen Lou Cang Jin’ |
| 14 | ‘Fen Mian Tao Sai’ |
| 15 | ‘Fen Xi Shi’ |
| 16 | ‘Feng Chu’ |
| 17 | ‘Gao Yuan Sheng Huo’ |
| 18 | ‘Gui Chuan Mei Hong’ |
| 19 | ‘Gui Chuan Yan Xia’ |
| 20 | ‘Han Hai Bing Xin’ |
| 21 | ‘Hei Tian Dong’ |
| 22 | ‘Hong Guan Yu Zan’ |
| 23 | ‘Hong Xian Nv’ |
| 24 | ‘Hong Xing’ |
| 25 | ‘Huang Yun’ |
| 26 | ‘Jin N20’ |
| 27 | ‘Jin N23’ |
| 28 | ‘Jin N28’ |
| 29 | ‘Jin Ye Fen’ |
| 30 | ‘Jing Bian Hua Hong’ |
| 31 | ‘Jing Cheng Zi’ |
| 32 | ‘Jing Di Hong’ |
| 33 | ‘Jing Die Man Wu’ |
| 34 | ‘Jing Duan Hong’ |
| 35 | ‘Jing Fei Hong’ |
| 36 | ‘Jing Fen Bai Yu Xin’ |
| 37 | ‘Jing Fen Chao Xia’ |
| 38 | ‘Jing Fen Fei Wu’ |
| 39 | ‘Jing Fen Guan Cai’ |
| 40 | ‘Jing Fen Lan’ |
| 41 | ‘Jing Feng Lan’ |
| 42 | ‘Jing Gou Zi’ |
| 43 | ‘Jing Guan Zi’ |
| 44 | ‘Jing Guang Hong’ |
| 45 | ‘Jing Gui Xiang Cui’ |
| 46 | ‘Jing Gui Zi’ |
| 47 | ‘Jing He Fen’ |
| 48 | ‘Jing He Gui’ |
| 49 | ‘Jing He Lan’ |
| 50 | ‘Jing He Wo Xue’ |
| 51 | ‘Jing He Zi’ |
| 52 | ‘Jing Hong Cha Cui’ |
| 53 | ‘Jing Hong Dian Cui’ |
| 54 | ‘Jing Hong Dian Jin’ |
| 55 | ‘Jing Hong Fei He’ |
| 56 | ‘Jing Hong Pi Shuang’ |
| 57 | ‘Jing Hong Ying Bo’ |
| 58 | ‘Jing Hong’ |
| 59 | ‘Jing Hua Mo Guan’ |
| 60 | ‘Jing Hua Zi’ |
| 61 | ‘Jing Jiao Fen’ |
| 62 | ‘Jing Jiao Hong’ |
| 63 | ‘Jing Jiao Zi’ |
| 64 | ‘Jing Ju Fen’ |
| 65 | ‘Jing Ju Fen’ |
| 66 | ‘Jing Juan’ |
| 67 | ‘Jing Li Zi’ |
| 68 | ‘Jing Ling Fen’ |
| 69 | ‘Jing Long Chun Hui’ |
| 70 | ‘Jing Long Fei Se’ |
| 71 | ‘Jing Long Wang Yue’ |
| 72 | ‘Jing Long Yao Hui’ |
| 73 | ‘Jing Long Zi’ |
| 74 | ‘Jing Mei Lan’ |
| 75 | ‘Jing Men Ma Nao Hong’ |
| 76 | ‘Jing Men Yan Hui’ |
| 77 | ‘Jing Mo Dan Xin’ |
| 78 | ‘Jing Mo Rui’ |
| 79 | ‘Jing Mo Sa Jin’ |
| 80 | ‘Jing Peng Zhan Chi’ |
| 81 | ‘Jing Rong Dong Bian’ |
| 82 | ‘Jing Shan Ri Hui’ |
| 83 | ‘Jing Shan Xi Zhao’ |
| 84 | ‘Jing Shan Yun Xiu’ |
| 85 | ‘Jing Shun Fen’ |
| 86 | ‘Jing Su Fen’ |
| 87 | ‘Jing Tao Hong’ |
| 88 | ‘Jing Tong Hong’ |
| 89 | ‘Jing Xi Zi’ |
| 90 | ‘Jing Xiu Gui’ |
| 91 | ‘Jing Xiu’ |
| 92 | ‘Jing Xiu’ |
| 93 | ‘Jing Xue Juan’ |
| 94 | ‘Jing Xue Qi Hui’ |
| 95 | ‘Jing Xue Ying Hui’ |
| 96 | ‘Jing Yan Fei’ |
| 97 | ‘Jing Yan Fen’ |
| 98 | ‘Jing Yan Hong’ |
| 99 | ‘Jing Yan Hong’ |
| 100 | ‘Jing Yan Luo Xue’ |
| 101 | ‘Jing Yan Ta Xue’ |
| 102 | ‘Jing Yan Zi’ |
| 103 | ‘Jing You Man’ |
| 104 | ‘Jing You Tu’ |
| 105 | ‘Jing Yu Dai’ |
| 106 | ‘Jing Yu Dan’ |
| 107 | ‘Jing Yu Fen’ |
| 108 | ‘Jing Yu Hong’ |
| 109 | ‘Jing Yu Hui’ |
| 110 | ‘Jing Yu Ling Long’ |
| 111 | ‘Jing Yu Sheng Hui’ |
| 112 | ‘Jing Yu Tian Cheng’ |
| 113 | ‘Jing Yu Xiang’ |
| 114 | ‘Jing Yu Xiu’ |
| 115 | ‘Jing Yuan Hong’ |
| 116 | ‘Jing Yue Lan’ |
| 117 | ‘Jing Yue Man’ |
| 118 | ‘Jing Yun Guan’ |
| 119 | ‘Jing Yun Hao’ |
| 120 | ‘Jing Yun Juan’ |
| 121 | ‘Jing Yun Xi’ |
| 122 | ‘Jing Yun Xiang’ |
| 123 | ‘Jing Yun Xiu’ |
| 124 | ‘Jing Zui Mei’ |
| 125 | ‘Jiu Nian Bai’ |
| 126 | ‘Lan Hai Yin Lang’ |
| 127 | ‘Lan Xian Nv’ |
| 128 | ‘Li Ren Zhuang’ |
| 129 | ‘Lian Tai’ |
| 130 | ‘Mu Ai’ |
| 131 | ‘Mu Chun Bai’ |
| 132 | ‘Qiang Wei Bai’ |
| 133 | ‘Qing Xin Hong’ |
| 134 | ‘Sha Mo Yan’ |
| 135 | ‘Sheng Hua Fen’ |
| 136 | ‘Shu Sheng Peng Mo’ |
| 137 | ‘Shuo Hei Dao Bai’ |
| 138 | ‘Tao Hua Xiang Yu’ |
| 139 | ‘Wei Zhi B-1’ |
| 140 | ‘Xing Gao Cai Lie’ |
| 141 | ‘Yan Shan Xue’ |
| 142 | ‘Yu Ban Xiu Qiu’ |
| 143 | ‘Yu Guan Yue’ |
| 144 | ‘Yu Pan Zhen Xiu’ |
| 145 | ‘Yu Pan’ |
| 146 | ‘Yu Rong Bing Xin’ |
| 147 | ‘Yu Zi Die’ |
| 148 | ‘Zi Die Ying Feng’ |
| 149 | ‘Zi Guan Yu Dai’ |
| 150 | ‘Zi Hai Yin Bo’ |
| 151 | ‘Zi Lou Xiang Yu’ |
| 152 | ‘Zi Lou Yan’ |
| 153 | ‘Zi Que She’ |
| 154 | ‘Zi Zhu Sha’ |
| 155 | ‘167#’ |
| 156 | ‘181-1#’ |
| 157 | ‘346#’ |
| 158 | ‘364#’ |
| 159 | ‘367#’ |
| 160 | ‘49#’ |

The names by numbers or codes refers to the accessions with their names in pending.

**Supplementary Table 2.** The 19 investigation traits and measurement standard.

| **Code** | **Trait** | | **Measurement standard** |
| --- | --- | --- | --- |
| 1 | Flower traits | Flower diameter | The width of flower at full bloom (cm) |
| 2 |  | Petal length | The length of the outer petal (mm) |
| 3 |  | Petal width | The width of the outer petal (mm) |
| 4 |  | Flare length | The flare length of the outer petal (mm) |
| 5 |  | Flare width | The flare width of the outer petal (mm) |
| 6 |  | Petal number | The number of petals in the whole flower |
| 7 |  | Carpel number | The number of carpels in the whole flower |
| 8 | Fruit traits | Number of carpels with seeds | The number of carpels that produce seeds in aggregate follicles |
| 9 |  | Multiple fruit fresh weight | The fresh weight of aggregate follicles (g) |
| 10 |  | Single fruit length | The length of one carpel in aggregate follicles (mm) |
| 11 |  | Single fruit width | The width of one carpel in aggregate follicles (mm) |
| 12 |  | Single fruit height | The height of one carpel in aggregate follicles (mm) |
| 13 |  | Single fruit pericarp thickness | The pericarp thickness of one carpel in aggregate follicles (mm) |
| 14 |  | Multiple fruit seed number | The number of seeds in aggregate follicles |
| 15 |  | Multiple fruit seed fresh weight | The weight of seeds in aggregate follicles (g) |
| 16 |  | Individual fruit number | The number of aggregate follicles in individual plant |
| 17 |  | Individual seed number | The number of seeds in individual plant |
| 18 |  | Individual fruit fresh weight | The weight of aggregate follicles in individual plant (g) |
| 19 |  | Individual seed fresh weight | The weight of seeds in individual plant (g) |

**Supplementary Table 3.** Information of 81 polymorphic EST-SSR used in this study.

| **Primer name** | **Primer sequence (5'-3')** | | **Repeat motif** | | **Expected size (bp)** | | **Annealing temperature (℃)** | |
| --- | --- | --- | --- | --- | --- | --- | --- | --- |
| PS2 | F | GCTCGTAGCTCTCACTGTCTGA | | (GA)6 | | 194 | | 60.4 |
|  | R | CTTCTCTTGGCGAAGGTCAC | |  | |  | |  |
| PS7 | F | CGTCAAGACTTATTCTTACCGTGTT | | (A)10 | | 297 | | 58.3 |
|  | R | AACCAACCATGGCGACAC | |  | |  | |  |
| PS8 | F | AACCTCGACGAATCAACG | | (T)10 | | 256 | | 57.5 |
|  | R | CGATGCCGACGGTCTAGTAT | |  | |  | |  |
| PS10 | F | GTCTCTGCTCATCGTGTCCA | | (T)13 | | 292 | | 59.9 |
|  | R | ACGCTGCCTCTCCTTCATT | |  | |  | |  |
| PS12 | F | CACTCTCTCCTCCTGCCAAG | | (CT)11 | | 200 | | 59.6 |
|  | R | TATCTTCCTCGGCTGTCCAT | |  | |  | |  |
| PS17 | F | AATACGGCACTGCAGCTTCT | | (T)11 | | 198 | | 58.3 |
|  | R | AAGAACCTAACCTAGACAATCTTCC | |  | |  | |  |
| PS19 | F | CCTCAGTCTTCACCTCTTCTCTC | | (A)13 | | 173 | | 56.7 |
|  | R | CTGGTACCACTTCCAACACG | |  | |  | |  |
| PS21 | F | TGGACCGGAAGATATGAAGC | | (TC)10 | | 282 | | 57.8 |
|  | R | GGCGGCACATCTGATTAAGT | |  | |  | |  |
| PS24 | F | TTGAGCAATCAGGTTCATTAGG | | (CAA)5 | | 155 | | 56.4 |
|  | R | TAGCCTCCGGTTCTGAATTG | |  | |  | |  |
| PS25 | F | GGCGTTGAATCATGTGTTCC | | (T)14 | | 279 | | 55.6 |
|  | R | CGTGAATGAGCGACTGCATA | |  | |  | |  |
| PS27 | F | CGCAGACTAGAATGCACCTG | | (A)12 | | 136 | | 52.0 |
|  | R | GGTTGGCTACTATCAATACAAGGA | |  | |  | |  |
| PS30 | F | TGAGATAGAAGCGGCAAGGT | | (T)11 | | 217 | | 56.3 |
|  | R | TAGCAGCGGCGAATATCTCT | |  | |  | |  |
| PS31 | F | TCCGGTGGACGATATTGATT | | (T)16 | | 248 | | 56.0 |
|  | R | GACAACAACAACATCGAAGTGA | |  | |  | |  |
| PS33 | F | CAGGACGACTACCTGCGTTA | | (GT)6(AT)10 | | 207 | | 56.7 |
|  | R | TCTTCCTGGCAATCTGAGGT | |  | |  | |  |
| PS36 | F | TCCAAGCTACTCCATGCCTTA | | (TCT)5 | | 277 | | 58.8 |
|  | R | GAATACTCACTCGCGGCTTC | |  | |  | |  |
| PS43 | F | TTCCTGCACTTAATCCAGTAGG | | (AG)10AAAGGAATTGGGAGCGAGAGAGGAATTGGG(GA)8 | | 239 | | 56.7 |
|  | R | GGCCGCATGCTATTAATGTT | |  |  |  | |  |
| PS46 | F | TCCAGGTATCTCCTGTCATTAACAT | | (GA)7 | | 210 | | 57.8 |
|  | R | GGATCTGGTAGAAGAGTTGCTG | |  | |  | |  |
| PS47 | F | TCTCAGCTTCTAATCTTCTCCTCA | | (AG)6 | | 246 | | 57.5 |
|  | R | ATGTCATGCCTCCAATCTCC | |  | |  | |  |
| PS49 | F | CATGCAATCTGCTTCTCAGG | | (T)20 | | 267 | | 56.5 |
|  | R | CTGATGCAGGATGCAATGTT | |  | |  | |  |
| PS50 | F | TTACAGCAGGCCACGACTG | | (AGC)6 | | 262 | | 55.9 |
|  | R | CATGACATCATGTGGTCCAA | |  | |  | |  |
| PS53 | F | CCGCCTTCTAAGGAGGATTC | | (A)13 | | 275 | | 53.2 |
|  | R | CCAGAAGCACCTGTGTATGGA | |  | |  | |  |
| PS55 | F | GGCAATGGCAACTGTATGAG | | (A)12 | | 265 | | 56.0 |
|  | R | CAGCAATTACAACTGTTCATGG | |  | |  | |  |
| PS56 | F | TTCGACCATCCATAAGTTGG | | (T)11 | | 276 | | 56.5 |
|  | R | AAGACAGGTTGCCTCGATTG | |  | |  | |  |
| PS57 | F | GCGACAGTACATTCCATCAA | | (TC)7 | | 128 | | 57.7 |
|  | R | GTCAACCACACGTCTGCAAG | |  | |  | |  |
| PS59 | F | CTTGATACCTGTCCGCCATT | | (GAA)5 | | 205 | | 56.6 |
|  | R | CATAACCATGAGCCATTCAAGA | |  | |  | |  |
| PS62 | F | GAACAAGGCAAGGTTGGTGT | | (T)10CTTCTCTTGTTTGATTGTCTTTGTTGTTATCT(GA)7 | | 225 | | 55.9 |
|  | R | TTACCAGGCAGTACTGAAGTTG | |  |  |  | |  |
| PS64 | F | GATTCTGTCTGGCATTGACG | | (GA)6 | | 293 | | 58.1 |
|  | R | CCATCTGTCTGGATCGACCT | |  | |  | |  |
| PS66 | F | GGAATTCTTGACCGCTCTTC | | (A)10GGTTTAGTTGAATTCACCATCCATCTTTAGCTAC(T)12 | | 221 | | 57.9 |
|  | R | CGTCAACCAACTGGTACGTC | |  |  |  | |  |
| PS73 | F | TGTCTGTACTTCATGACAGAACCTC | | (T)11 | | 251 | | 59.3 |
|  | R | ACAGCCAAGAACATCCAGGT | |  | |  | |  |
| PS75 | F | TTGAACAGGCAATTCAGTGG | | (C)16 | | 271 | | 56.7 |
|  | R | CGCTTCCACCACTACCAATA | |  | |  | |  |
| PS85 | F | GGTGAAGATGATTATGGCTTCC | | (A)10 | | 275 | | 56.3 |
|  | R | CATTCATTAGCACCACCTAACC | |  | |  | |  |
| PS90 | F | CACGTCATGGCAATGTGAA | | (T)15 | | 251 | | 57.2 |
|  | R | TCCATAGAGTGGCAACATGC | |  | |  | |  |
| PS91 | F | GTGGATCCAGAGGTTCCTGA | | (T)15 | | 283 | | 59.5 |
|  | R | AGTGCCATAGAGTGGCAACA | |  | |  | |  |
| PS93 | F | TGCTGCAAGATGAACTCCAG | | (T)11 | | 122 | | 57.3 |
|  | R | TCTCTCAACAAGCAAGAACTCA | |  | |  | |  |
| PS94 | F | TCGATCTATATCCATGGCTCAC | | (TC)8 | | 255 | | 57.4 |
|  | R | AGTCTCTCTCCTTGCTTGGAA | |  | |  | |  |
| PS96 | F | AAGAGTAACATGCGCCAACA | | (CT)18 | | 242 | | 58.8 |
|  | R | AACACTACAAGACCTCCACTGC | |  | |  | |  |
| PS97 | F | ATTGCCGTATTCCTCGTGAC | | (T)11 | | 181 | | 52.9 |
|  | R | CCACGTGCTACTACACTATGCTT | |  | |  | |  |
| PS98 | F | CAGCAACAACCGATAATGGA | | (CAA)5 | | 289 | | 51.7 |
|  | R | CATCCACTTCCTCGTCGTCT | |  | |  | |  |
| PS102 | F | TGAGTGAACTCCTCCTCCTTG | | (T)11 | | 282 | | 59.0 |
|  | R | CCACTAGATGAAGCCATGGTG | |  | |  | |  |
| PS103 | F | AAGATGCAGAGGATCGAGGA | | (T)15 | | 214 | | 58.1 |
|  | R | CATGTCGAAGCACTGTAACCA | |  | |  | |  |
| PS105 | F | TTCATATCGGACACGCAGAG | | (GTA)5 | | 279 | | 59.0 |
|  | R | AGTTCTGCACTCGAGCTTCC | |  | |  | |  |
| PS113 | F | CATTAGCTCAGCTGCCTCAG | | (T)16 | | 259 | | 53.7 |
|  | R | GCGACTGACATTCTGACTCGT | |  | |  | |  |
| PS114 | F | TCCATCATCATCGTCATCAGA | | (T)14 | | 287 | | 48.7 |
|  | R | GCCAATAATGGCAACAACA | |  | |  | |  |
| PS116 | F | GACTTGCACTCGAACCATCA | | (A)10 | | 190 | | 57.6 |
|  | R | CATAATCTCCGCCGGTTCTA | |  | |  | |  |
| PS117 | F | CATAATCTCCGCCGGTTCTA | | (T)11 | | 190 | | 57.6 |
|  | R | GACTTGCACTCGAACCATCA | |  | |  | |  |
| PS118 | F | GGATTACATGTGAAGCGGATG | | (TC)6 | | 290 | | 58.2 |
|  | R | GCGCCACAGACTTCAATACC | |  | |  | |  |
| PS122 | F | TTGCTCCTAGGTATCGCAATC | | (T)12 | | 295 | | 57.0 |
|  | R | AAGGACGTATTGACGCAGAA | |  | |  | |  |
| PS123 | F | TTCAGCAGCAACAACAACAA | | (ACA)5 | | 245 | | 55.9 |
|  | R | CTGAGCGAGAACCACCAGTT | |  | |  | |  |
| PS129 | F | GATTCTCCATGCCATTGTCC | | (T)13 | | 168 | | 57.3 |
|  | R | CAGCCACCTTCATGCTGATA | |  | |  | |  |
| PS131 | F | CAACCTTGGTCTTGCTCTCC | | (GAA)6 | | 210 | | 57.7 |
|  | R | GCTCTAATCCTCGAATATTACCTGA | |  | |  | |  |
| PS142 | F | TCCAAGATGAGAAGTGCATCC | | (A)10 | | 218 | | 58.6 |
|  | R | AGCAACTGCGGAAGATGAGT | |  | |  | |  |
| PS145 | F | CAGTGGATCATCGCACAATC | | (T)10 | | 258 | | 57.1 |
|  | R | AAGACCAGCCAATGAACCAA | |  | |  | |  |
| PS147 | F | GGCCTCTCTGCAGTCGTTA | | (T)12 | | 145 | | 56.2 |
|  | R | GACGACTAGCAACTATTCCACAA | |  | |  | |  |
| PS151 | F | GCCACACCTCACTTGGTCTT | | (T)13 | | 210 | | 59.1 |
|  | R | AGGCGCAATTCTCATGTCTT | |  | |  | |  |
| PS159 | F | TTATGGAGCAGTGATGCTTCT | | (T)13 | | 245 | | 57.7 |
|  | R | ATCCACCTGCCTGTCTGATT | |  | |  | |  |
| PS160 | F | ATGCACCTGTGGAGGTGTG | | (T)13 | | 265 | | 60.0 |
|  | R | CAAGGTACTACGCGAACCTG | |  | |  | |  |
| PS163 | F | CTCGAGCAAGAGTTGGTGGT | | (TC)6CT(CA)7 | | 276 | | 61.0 |
|  | R | ACAGGAGTCTCGCCTAGCAA | |  | |  | |  |
| PS002 | F | TCGGTGACCTGGCTGTTG | | (GA)8 | | 241 | | 58.0 |
|  | R | CCCTCCTCCCACTTTGTT | |  | |  | |  |
| PS026 | F | TTCCCTCCATTCTAACAC | | (AG)6 | | 187 | | 54.0 |
|  | R | ACCCTAGCCTCTGACATT | |  | |  | |  |
| P061 | F | CTCCTCCAACATTGACCC | | (TG)8 | | 154 | | 57.0 |
|  | R | CACCCTCCCAAACATCTC | |  | |  | |  |
| P067 | F | TGCCTAGCTTACCACATC | | (GA)6 | | 462 | | 51.0 |
|  | R | CAAGGGAATTTCTTTCTG | |  | |  | |  |
| P068 | F | CTTTGGCATTCTCATTCA | | (TC)7 | | 174 | | 52.5 |
|  | R | GGTGGTATTGGGCTTCTT | |  | |  | |  |
| P138 | F | CCTCACTTCCTCCACCAC | | (CCG)5 | | 330 | | 58.5 |
|  | R | GTTCCGTTCGTACCCTTC | |  | |  | |  |
| P150 | F | CTCTGGTCATCCGTAAGC | | (CCA)5 | | 365 | | 55.0 |
|  | R | AGGAAGAAGTAAAGTGGGT | |  | |  | |  |
| P162 | F | CAGTCTTTCATGGCTTCAC | | (AG)6 | | 357 | | 51.0 |
|  | R | CATTTATTTGCGTGTTCC | |  | |  | |  |
| P180 | F | CCCCGAAATGGAGGAGTC | | (CT)6 | | 188 | | 60.0 |
|  | R | AGGGCAGTAGCAGAAGAAAGTC | |  | |  | |  |
| P221 | F | GATACAAGGCGGAAAGTG | | (AAT)5 | | 301 | | 56.0 |
|  | R | AGAGTTGGGAACCAGACC | |  | |  | |  |
| P235 | F | CGCATCTTTCAACTCACG | | (GAG)5 | | 265 | | 51.5 |
|  | R | TCACCCAGTCACTTAAATACC | |  | |  | |  |
| P242 | F | TCTAAGCCAGAGCCCAAGT | | (TCTCGT)3 | | 199 | | 57.5 |
|  | R | CCAAGCCCAACCAAAGTAC | |  | |  | |  |
| P260 | F | ATTCACGCCAGTATCAAAG | | (CCTGGA)3 | | 349 | | 53.0 |
|  | R | TGTAAATGCCCATGTCTAG | |  | |  | |  |
| P265 | F | TTTTATGGGTCCTGTTGC | | (ACAGCC)4 | | 290 | | 54.0 |
|  | R | GAAGAGTAAGCCTTTGTCG | |  | |  | |  |
| P280 | F | CATCCCTGAGTGGTGTAAT | | (CGAAAT)3 | | 377 | | 52.5 |
|  | R | TTGTCGTCCTTTCTTGTT | |  | |  | |  |
| P281 | F | AGACCTTTTAGAGCAGTA | | (CTCTTT)3 | | 150 | | 50.5 |
|  | R | TCAGATAAGAGTCCTTTC | |  | |  | |  |
| P290 | F | TTCTTTCACCTCCACTTCA | | (AGTAGG)4 | | 245 | | 53.0 |
|  | R | CGTCGTTTCGTTTACTCTT | |  | |  | |  |
| P296 | F | CTCTTTCGCTGCCACAAC | | (GAAGCA)4 | | 419 | | 57.5 |
|  | R | CTCTGCTCTTCCCGTCTT | |  | |  | |  |
| P318 | F | ATCTACTCCCTATCCGTCAC | | (CCACA)4 | | 369 | | 55.0 |
|  | R | CCCACAGCTACCTCCTTG | |  | |  | |  |
| P333 | F | AAGCACGGTGTTCCAGAT | | (TCACCA)3 | | 415 | | 52.5 |
|  | R | ACGGGCTCCTTACCACAT | |  | |  | |  |
| Seq6 | F | GACCGATTTGACCCTCTA | | (GA)11 | | 219 | | 52.5 |
|  | R | CTCCCATGTGATGTTGTG | |  | |  | |  |
| 50F, R | F | AGAAGAGTAACATGCGCC | | (CT)10 | | 380 | | 52.0 |
|  | R | AAGACCTCCACTGCAGAT | |  | |  | |  |
| 5F, R | F | CGTTCAATCTATGTCGTACC | | (ACC)5 | | 400 | | 53.0 |
|  | R | TGCTCATCTACTACAACAGG | |  | |  | |  |
| PCA1 | F | TAGTCAGTCGTAGCTAGCATAGGCA | | (GT)20 | | 142 | | 60.0 |
|  | R | GATGGCCACCTATAGAAAAGAATC | |  | |  | |  |

# Supplementary Figures

**
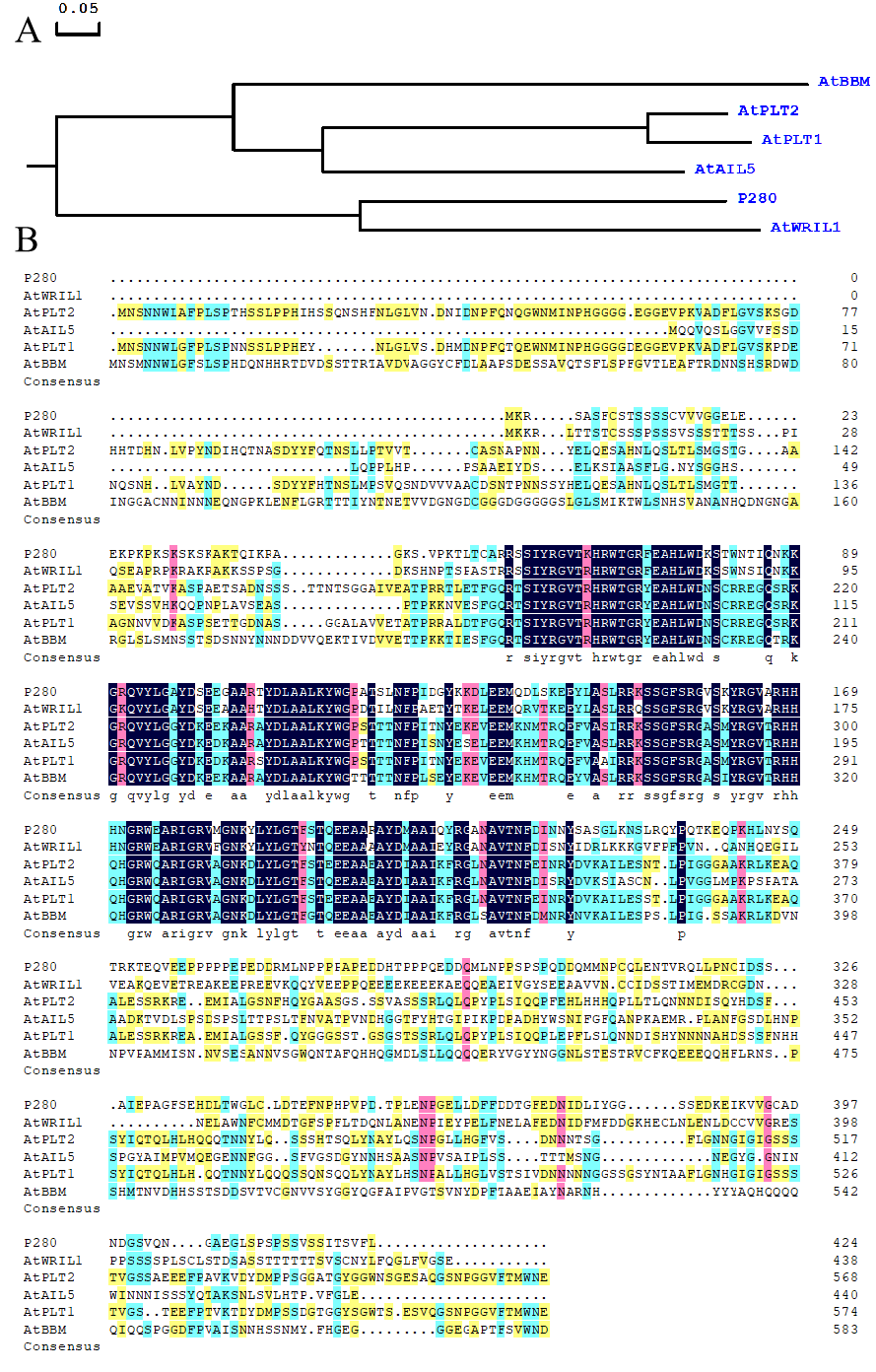
**

**Supplementary Figure 1.** Analysis results of P280 predicted protein and homologous proteins. (A) Phylogenetic tree; (B) Sequence alignment map.

Note: PS280 (GBGY01000124.1), AtWRIL1 (AT3G54320.3; NP_001030857.1), AtPLT2 (AT1G51190.1; NP_175530.2), AtAIL5 (AT5G57390.1; NP_001331405.1), AtPLT1 (AT3G20840.1; NP_188720.2), AtBBM (AT5G17430.1; NP_001332647.1).


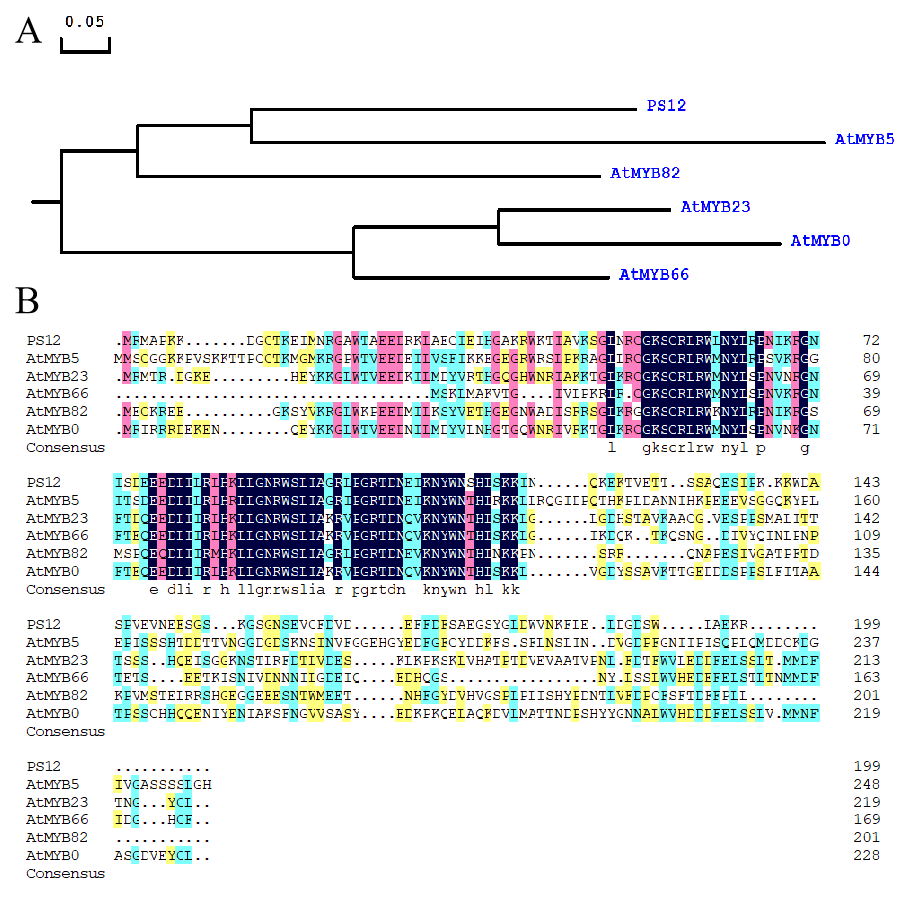


**Supplementary Figure 2.** Analysis results of PS12 predicted protein and homologous proteins. (A) Phylogenetic tree; (B) Sequence alignment map.

Note: PS12 (PB.59960.1), AtMYB5 (AT3G13540.1; NP_187963.1), AtMYB23 (AT5G40330.1; NP_198849.1), AtMYB66 (AT5G14750.1; NP_001331609.1), AtMYB82 (AT5G52600.1; NP_680426.1), AtMYB0 (AT3G27920.1; NP_189430.1).

**
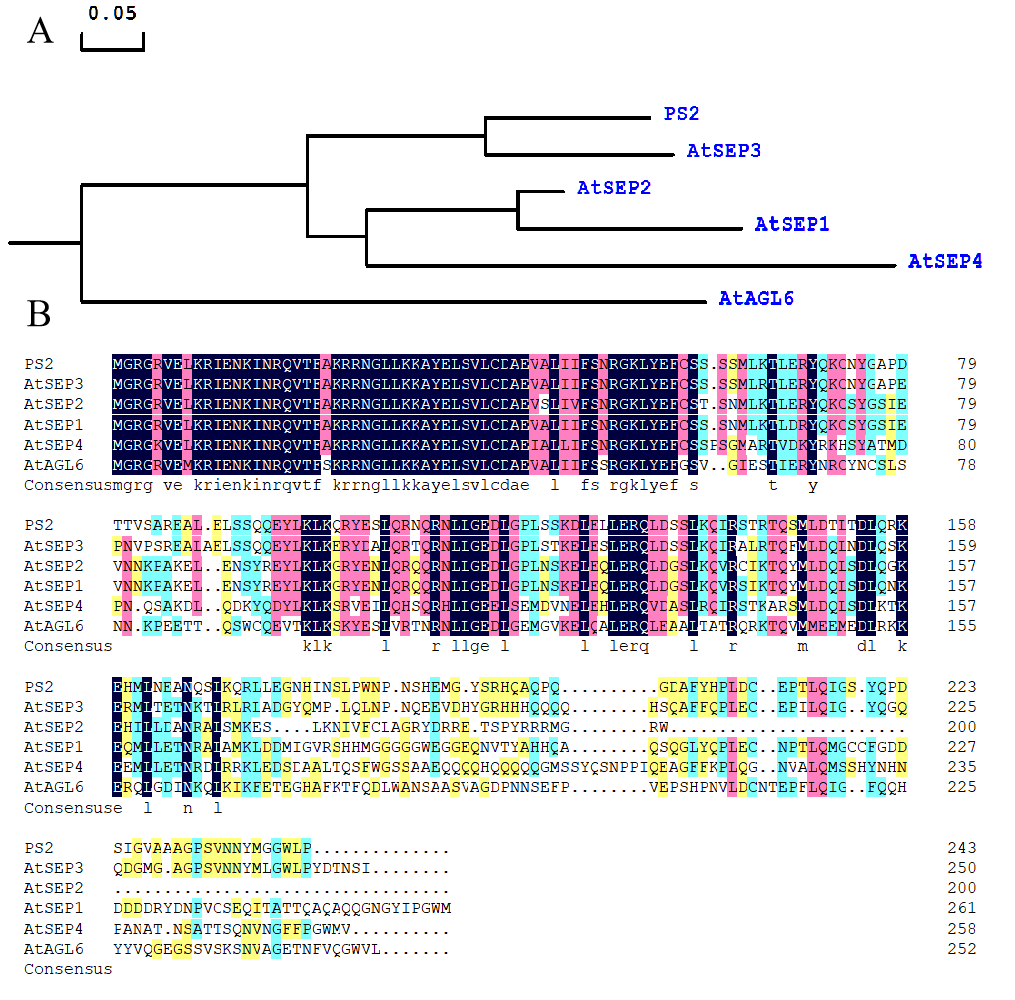
**

**Supplementary Figure 3.** Analysis results of PS2 predicted protein and homologous proteins. (A) Phylogenetic tree; (B) Sequence alignment map.

Note: PS2 (PB.55834.2), AtSEP3 (AT1G24260.1; NP_001185081.1), AtSEP2 (NP_001325746.1; AT3G02310.1), AtSEP1 (AT5G15800.1; NP_001119230.1), AtSEP4 (AT2G03710.2; NP_178466.1), AtAGL6 (AT2G45650.1; NP_182089.1).


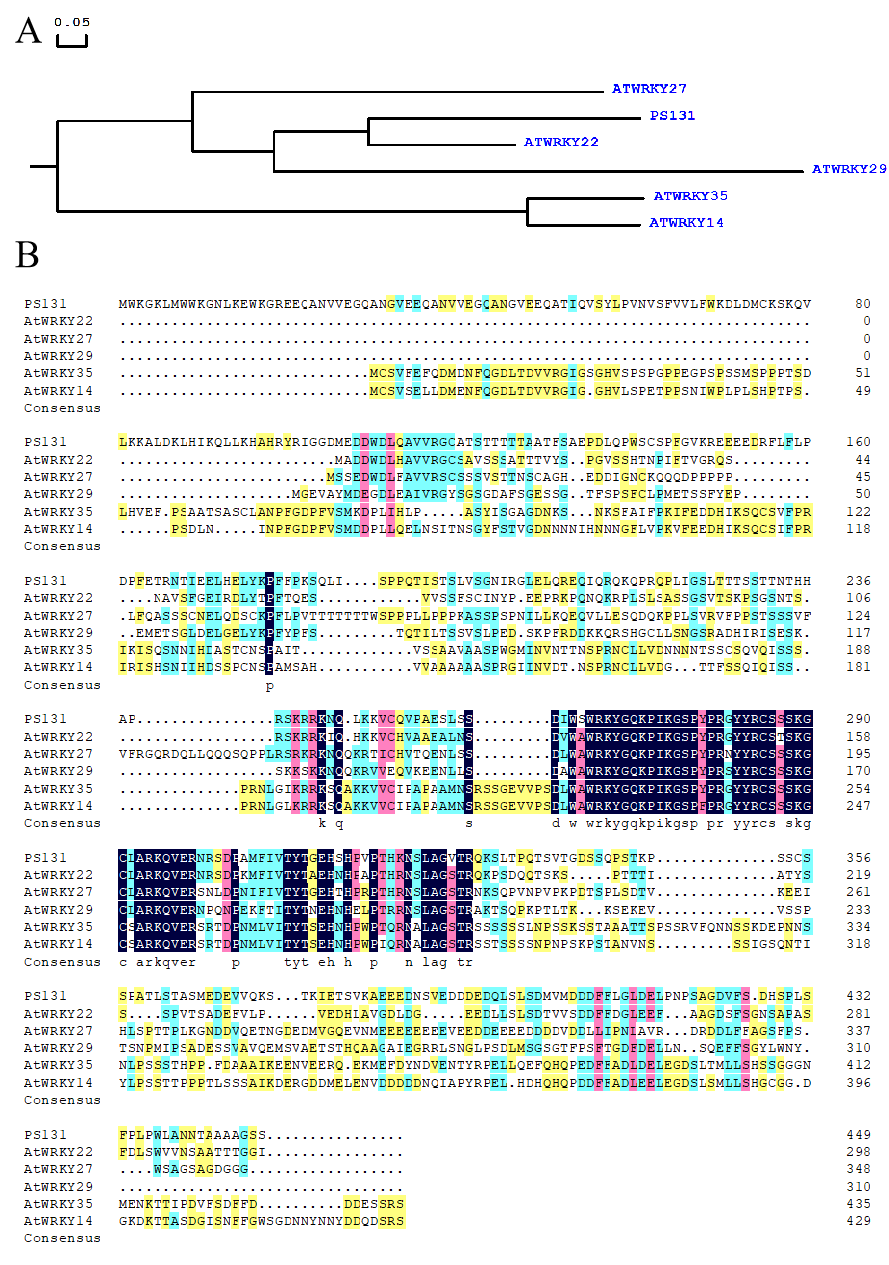


**Supplementary Figure 4.** Analysis results of PS131 predicted protein and homologous proteins. (A) Phylogenetic tree; (B) Sequence alignment map.

Note: PS131 (PB.52783.1), AtWRKY22 (AT4G01250.1; NP_192034.1), AtWRKY27 (AT5G52830.1; NP_568777.1), AtWRKY29 (AT4G23550.1; NP_194086.4), AtWRKY35 (AT2G34830.2; NP_001324223.1), AtWRKY14 (AT1G30650.1; NP_564359.1).


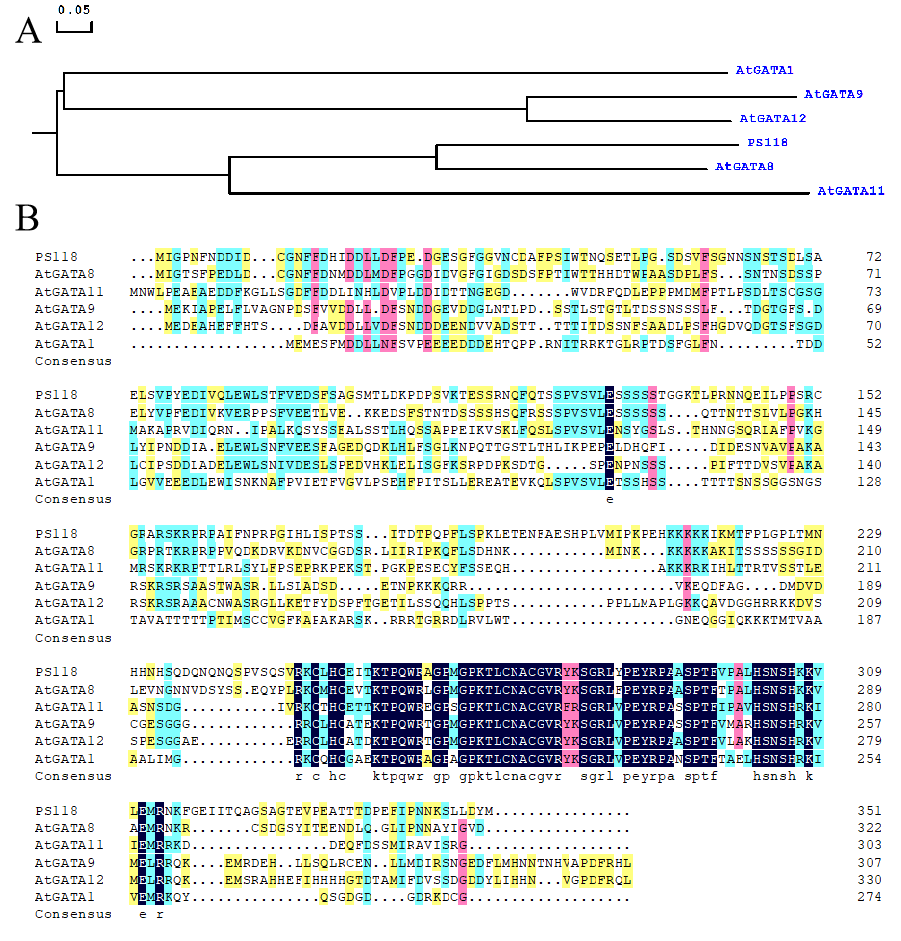


**Supplementary Figure 5.** Analysis results of PS118 predicted protein and homologous proteins. (A) Phylogenetic tree; (B) Sequence alignment map.

Note: PS118 (PB.45875.1), AtGATA8 (AT3G54810.2; NP_191041.1), AtGATA11 (AT1G08010.3; NP_001077485.1), AtGATA9 (AT4G32890.1; NP_195015.1), AtGATA12 (AT5G25830.1; NP_197955.1), AtGATA1 (AT3G24050.1; NP_189047.1).


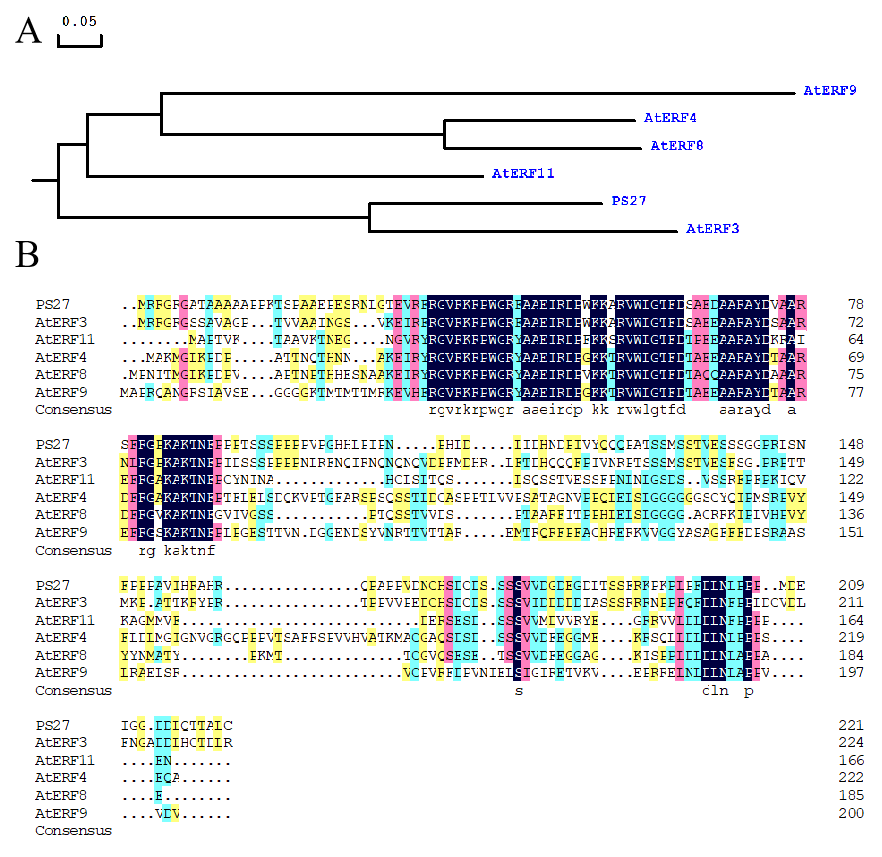
**Supplementary Figure 6.** Analysis results of PS27 predicted protein and homologous proteins. (A) Phylogenetic tree; (B) Sequence alignment map.

Note: PS27 (PB.60194.1), AtERF3 (AT1G50640.1; NP_175479.1), AtERF11 (AT1G28370.1; NP_001319098.1), AtERF4 (AT3G15210.1; NP_188139.1), AtERF8 (AT1G53170.1; NP_175725.1), AtERF9 (AT5G44210.1; NP_199234.1).
